# Screening for post-TB lung disease at TB treatment completion: Are symptoms sufficient?

**DOI:** 10.1371/journal.pgph.0002659

**Published:** 2024-01-29

**Authors:** Jamilah Meghji, Vester Gunsaru, Beatrice Chinoko, Elizabeth Joekes, Ndaziona P. K. Banda, Nicola Marozva, Jamie Rylance, Stephen B. Squire, Kevin Mortimer, Maia Lesosky

**Affiliations:** 1 National Heart & Lung Institute, Imperial College London, London, United Kingdom; 2 Malawi Liverpool Wellcome Clinical Research Programme, Blantyre, Malawi; 3 Department of Clinical Sciences, Liverpool School of Tropical Medicine, Liverpool, United Kingdom; 4 Department of Medicine, Kamuzu University of Health Sciences and Queen Elizabeth Central Hospital, Blantyre, Malawi; 5 Division of Epidemiology & Biostatistics, University of Cape Town, Cape Town, South Africa; 6 Department of Respiratory Medicine, Liverpool University Hospitals National Health Service Foundation Trust, Liverpool, United Kingdom; University of California Irvine, UNITED STATES

## Abstract

Pulmonary TB survivors face a high burden of post-TB lung disease (PTLD) after TB treatment completion. In this secondary data analysis we investigate the performance of parameters measured at TB treatment completion in predicting morbidity over the subsequent year, to inform programmatic approaches to PTLD screening in low-resource settings. Cohort data from urban Blantyre, Malawi were used to construct regression models for five morbidity outcomes (chronic respiratory symptoms or functional limitation, ongoing health seeking, spirometry decline, self-reported financial impact of TB disease, and death) in the year after PTB treatment, using three modelling approaches: logistic regression; penalised regression with pre-selected predictors; elastic net penalised regression using the full parent dataset. Predictors included demographic, clinical, symptom, spirometry and chest x-ray variables. The predictive performance of models were examined using the area under the receiver-operator curve (ROC AUC) values. Key predictors were identified, and their positive and negative predictive values (NPV) determined. The presence of respiratory symptoms at TB treatment completion was the strongest predictor of morbidity outcomes. TB survivors reporting breathlessness had higher odds of spirometry decline (aOR 20.5, 95%CI:3–199.1), health seeking (aOR 10.2, 2.4–50), and symptoms or functional limitation at 1-year (aOR 16.7, 3.3–133.4). Those reporting activity limitation were more likely to report symptoms or functional limitation at 1-year (aOR 4.2, 1.8–10.3), or severe financial impact of TB disease (aOR2.3, 1.0–5.0). Models were not significantly improved by including spirometry or imaging parameters. ROC AUCs were between 0.65–0.77 for the morbidity outcomes. Activity limitation at treatment completion had a NPV value of 78–98% for adverse outcomes. Our data suggest that whilst challenging to predict the development of post-TB morbidity, the use of symptom screening tools at TB treatment completion to prioritise post-TB care should be explored. We identified little benefit from the additional use of spirometry or CXR imaging.

## Introduction

There are over 10 million incident cases of tuberculosis (TB) each year [1]. Over 85% of TB patients successfully complete treatment, with an estimated 155 million TB survivors alive in 2020 [2], but many TB survivors continue to experience physical, psychological and socioeconomic morbidity long after treatment completion [3–7]. Post-TB lung disease (PTLD) is a well described sequela of TB disease, with approximately half of those successfully treated for pulmonary TB (PTB) in low- and middle-income countries (LMICs) left with respiratory symptoms, impaired spirometry, or structural lung damage [8–10]. Although there is some recovery over time, a minority of PTB survivors continue to experience morbidity even several years after TB treatment completion [11], and PTLD has been associated with functional impairment, loss of income, and ongoing health seeking [6,12,13].

In response to this emerging data on the burden of PTLD, several National TB Programmes (NTPs) have considered screening for PTLD at TB treatment completion [14], and the provision of ongoing clinical care to those with residual lung damage. However, there is a lack of data to inform screening approaches. It remains unclear whether symptoms, spirometry, or imaging could be used to identify patients with residual PTLD who are at risk of adverse outcomes. The costs and feasibility of these approaches have not been described. Similarly the potential impact of PTLD screening on patients is not well understood–benefits may include linkage to post-TB care and health education, but harms may include stigma related to a chronic disease diagnosis, and the time and cost associated with further investigation. There is a need for more robust evidence around screening to inform decision making by NTPs around implementation [15].

In this paper we perform a secondary analysis of published cohort data from Malawi [9], to investigate the performance of symptom questions, spirometry, and chest radiographs (CXR) at TB treatment completion in predicting adverse outcomes in the year after TB treatment completion, in order to inform programmatic approaches to PTLD screening in low-resource settings.

## Methods

This exploratory analysis used data from a prospective cohort study of 405 PTB-survivors in urban Blantyre, Malawi, which has been described in detail elsewhere [9]. Briefly, adults ≥15-years successfully completing treatment for a first episode of drug-sensitive PTB were sequentially recruited between February 2016 –April 2017. Respiratory symptoms, quality of life (St George’s Respiratory Questionnaire (SGRQ) and the EQ-5D-3L tool) and socioeconomic parameters were measured at TB treatment completion, and at 6- and 12-months thereafter. Participants completed questionnaires in the local language (Chichewa). Post-bronchodilator American Thoracic Society (ATS)-standard spirometry was performed at each study visit, and digital CXRs were completed at TB treatment completion. All-cause deaths were confirmed with family and friends, where participants failed to attend study follow-up visits. It was not possible to determine the cause of death. All CXRs were reported independently by both a pulmonologist (JM) and consultant radiologist (EJ) using a specifically designed reporting tool (S1 Text) with consensus review to generate final results.

### Data analysis

Adverse outcomes thought to be relevant to patient lives were defined by the study team (Table 1). The frequency and overlap between these outcomes were described. Change in spirometry over time was included as an outcome of interest, rather than absolute spirometry at 1-year, on the basis that the trajectory of decline may be particularly important to long-term outcomes. TB retreatment was strongly associated with death, and so was not examined separately.

**Table 1:** Post-TB outcomes of interest, determined at 1-year after TB treatment completion.

Outcome	Definition
All-cause mortality	Death confirmed by the participant’s family and friends
Accelerated spirometry decline*	Fall in FEV_1_ OR FVC by >100ml between TB treatment completion and 1-year after treatment completion
Respiratory health seeking †	≥1 unscheduled visit to health care provider, either outpatient or inpatient, due to a respiratory complaint (cough, breathlessness, sputum, wheeze, chest pain) in the 1-year after treatment completion
Symptoms or functional limitation‡	Self-reported presence of respiratory symptoms (cough, breathlessness, sputum, or wheeze) ≥ several days/week in the past three months OR limitation in routine activities due to chest symptoms, at 1-year after TB treatment completion
Severe financial impact ^¶^	Severe financial impact of TB-disease on the household, self-reported at 1-year after TB treatment completion

*Only participants with acceptable and usable spirometry data meeting QC standards at both TB treatment completion and 1-year included (n = 305). Threshold of 100ml based on minimal clinically important differences used in COPD literature [16,17].

^†^Self-reported health seeking in response to questions: “How many times have you visited a health care provider as an outpatient / have you been admitted to hospital since (you completed TB treatment/we last saw you)?”, and “Why did you go to see the health care provider?”, with verification in Health Passport where possible.

^‡^Self-reported symptoms occurring on ‘several’ or ‘most’ days post week in response to the St George’s Respiratory Questionnaire (SGRQ) questions for each symptom: “Over the past 3-months I have (coughed / brought up sputum / had shortness of breath / had attacks of wheezing)”, with response options including “Not at all /only with chest infections / a few days per month / several days per week / most days per week” OR.

Reported limitation of at least one or two activities, in response to the question “Please tell us which of these statements best describes how your chest affects you”, with response options including “It does not stop me doing anything I would like to do / It stops me doing one or two things / most of the things / everything I would like to do”.

^¶^Self-reported financial impact of 4–5 in response to Likert question: “On a scale of 1 to 5, in which 1 is no impact and 5 is very serious impact, to what extent has your TB illness affected the family financially, up until this time”.

Two approaches were used to select predictors and construct models for each outcome. Firstly, a parsimonious set of potential predictors were selected *a priori* by the study team (S2 Text) and models were constructed in a layered fashion with sequential inclusion of chosen demographic, clinical, spirometry, and finally CXR variables. Both logistic regression models and penalised regression models were applied to these pre-selected variable sets. Firth’s correction was used to obtain estimates for models using death as the outcome. Secondly, variable selection via elastic net penalised regression models was used to identify key predictors from amongst the larger set of parameters available within the parent data set (S3 Text). This approach was taken to evaluate if our *a priori* predictor sets were incomplete and missing highly predictive or relevant variables. For all penalised regression models, variable importance factors (VIF) were calculated and plotted. Selected variables under the best performing model were re-fit and estimated coefficients (95% CI) presented. All models were fit under 10-fold cross validation and model performance was visualised using area under the receiver-operator (ROC) curve (AUC). Only demographic, clinical, spirometry and CXR parameters which could reasonably be measured at TB treatment completion in LMICs were included. Missing data were imputed for socioeconomic status at TB treatment completion (n = 33), but complete case analyses were used elsewhere.

### Approvals & permissions

All participants provided written informed consent for the parent study. Ethical approval for the parent study was obtained from the Liverpool School of Tropical Medicine (15.040RS) and Malawi College of Medicine Research Ethics (P.10/15/1813) Committees. This study included a secondary analysis of an anonymised dataset by the original study team, and no additional approvals were therefore sought.

## Results

### Study population

As described previously, 405 participants were recruited. Median age was 35-years (interquartile range (IQR): 28–41), and the majority were male (67.9%, 275/405). Over three quarters had been treated for microbiologically proven pulmonary TB disease (77.3%, 313/405) and 60.2% (244/405) had HIV at TB treatment completion (Table 1). Over the 1-year follow up period, 11/405(2.7%) participants died, 25/405(6.2%) withdrew or relocated, and it was not possible to determine the final outcome of 1 participant only. A total of 368 participants completed the final 1-year study visit.^9^

### Prevalence of adverse patient outcomes

ATS standard spirometry was available for 305 adults at both TB treatment completion and 1-year after TB treatment completion, with accelerated decline observed in 23.7% (71/305). A total of 16.8% (62/368) of those who contributed at least 6-months of follow-up data had at least one unscheduled health seeking episode for a respiratory problem. Persistent symptoms or functional limitation were reported by 19.8% (73/368) at 1-year, and 16.8% (62/368) reported a severe financial impact of TB disease.

### Relationship between patient outcomes

**Fig 1:**
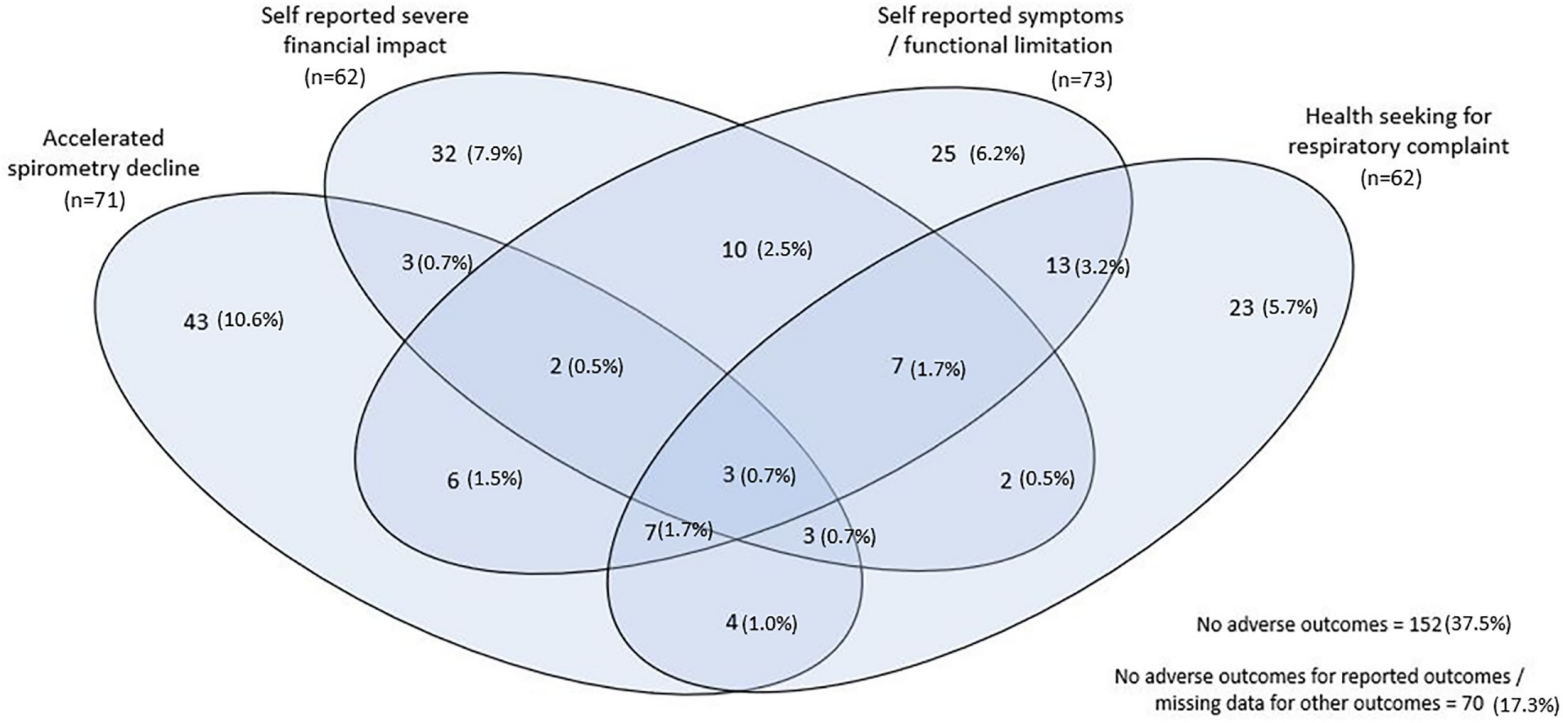
Overlap between patients experiencing adverse outcomes, in the 1-year after TB treatment completion, with proportion of the total study population (n = 405) given for each group.

The majority of participants experiencing any negative outcome had only a single adverse outcome (Spirometry decline alone 60.6% [43/71]; Severe financial impact alone 51.6% [32/62]; Chronic respiratory symptoms alone 34.2%[25/73]; Unscheduled health seeking alone 37.1% [23/62]). The greatest overlap in adverse outcomes was seen between those with chronic symptoms at 1-year and those reporting unscheduled health seeking over this year: 22.2%(30/135) of those with either of these outcomes experienced both (Fig 1).

### Characteristics by individual adverse outcomes, in univariate analysis

As previously described, the majority of those who died were HIV-positive (90.1%, 10/11), with CD4 counts below 200 cells/mm^3^ at TB treatment completion (80%, 8/10).^9^ The prevalence of limited walking pace, or ≥10% parenchymal consolidation on CXR at TB treatment completion was higher amongst those who subsequently died, compared to those who survived (54.5% vs. 26.0%, p = 0.035 and 27.3% vs. 7.1%, p = 0.013 respectively) (Table 2).

**Table 2:** Participant characteristics, for whole cohort and those experiencing individual outcomes, with univariate relationships between parameters and outcomes shown by density of shading (P<0.001, p<0.01, p<0.05).

Predictor, measured at TB treatment completion*	Total (n = 405)	Adverse outcome, over 1-year*				
		Death (n = 11/405)	Spirometry decline (n = 71/305)	Health seeking (n = 62/368)	Symptoms / limitation (n = 73/368)	Severe financial impact (n = 62/368)
Demographic parameters (n = 405)						
Age (yrs) (median, IQR)	35 (28–41)	41 (36–45.5)	33 (28–43.5)	35 (29–39)	34 (30–41)	39 (33–44)
Male sex (n, %)	275 (67.9%)	8 (72.7%)	42 (59.2%)	39 (62.9%)	48 (65.8%)	46 (74.2%)
Positive TB microbiology (n, %)†	313 (77.3%)	5 (45.5%)	55 (77.5%)	49 (79.0%)	55 (75.3%)	45 (72.6%)
HIV status (n = 403)						
• Negative	159 (39.5)	1 (9.1%)	31 (43.7%)	32 (51.6%)	34 (47.2%)	17 (27.4%)
• Positive, CD4<200	102 (25.3%)	8 (72.7%)	19 (26.8%)	11 (17.7%)	18 (25.0%)	21 (33.9%)
• Positive, CD4 > = 200	142 (35.2%)	2 (18.2%)	21 (29.6%)	19 (30.6%)	20 (27.8%)	24 (38.7%)
Maximum education level > primary school (n, %)	251 (62.0%)	9 (81.8%)	42 (59.2%)	38 (61.3%)	37 (50.7%)	30 (48.4%)
Poorest 2 socioeconomic quintiles (n, %)	107 (28.8%)	1 (25%)	22 (31.0%)	20 (32.8%)	24 (33.8%)	27 (44.3%)
Ever smoked (n, %)	120 (29.6%)	2 (18.2%)	20 (28.2%)	12 (19.4%)	25 (34.2%)	26 (41.9%)
Main fuel (n, %)						
• Charcoal	338 (83.5%)	8 (72.7%)	56 (78.9%)	53 (85.5%)	63 (86.3%)	51 (82.3%)
• Electricity	21 (5.2%)	2 (18.2%)	5 (7.0%)	1 (1.6%)	0	1 (1.6%)
• Wood	46 (11.4%)	1 (9.1%)	10 (14.1%)	8 (12.9%)	10 (13.7%)	10 (16.1%)
Clinical parameters at TB treatment completion (n = 405) ‡						
BMI (kg/m^2^) median (IQR)	20.5 (19.0–22.3)	19.8 (17.3–20.5)	21.3 (19.7–23.0)	20.6 (18.8, 21.8)	19.7 (18.6–21.7)	20.4 (18.6–21.9)
Weekly cough (n, %)	11 (2.7%)	0	1 (1.4%)	2 (3.2%)	5 (6.8%)	2 (3.2%)
Weekly breathlessness (n, %)	17 (4.2%)	1 (9.1%)	5 (7.0%)	7 (11.3%)	12 (16.4%)	1 (1.6%)
Limited walking pace (n = 403) (n, %)	108 (26.8%)	6 (54.5%)	16 (22.9%)	20 (32.3%)	36 (49.3%)	17 (27.9%)
Limitation of activities (n = 403) (n, %)	205 (50.6%)	8 (72.7%)	37 (52.1%)	40 (64.5%)	58 (79.5%)	38 (61.3%)
Spirometry at TB treatment completion (n = 365) ^¶^						
FEV_1_% predicted (median, IQR)	85.5 (73.2–98.3)	82.7 (78.5–93.4)	92.8 (83.4–105.8)	82.7 (69–92.8)	81.2 (65.3–90.7)	86.8 (76.6–93.6)
FVC % predicted (median, IQR)	88.6 (78.8–98)	89.1 (81–96.6)	96.9 (87.1–108)	87.9 (78.1–96.4)	89.2 (73.5–97.4)	90.4 (81–96.3)
Pattern						
• Normal	240 (65.8%)	9 (81.8%)	56 (78.9%)	15 (25.9%)	36 (57.1%)	9 (16.1%)
• Obstruction	52 (14.2%)	1 (9.1%)	10 (14.1%)	34 (58.6%)	14 (22.2%)	38 (67.9%)
• Low FVC	73 (20.0%)	1 (9.1%)	5 (7.0%)	9 (15.5%)	13 (20.6%)	9 (16.1%)
Chest X-Ray at TB treatment completion (n = 403)^						
Ring & tramline markings	119 (29.5%)	1 (9.1%)	16 (22.5%)	17 (27.4%)	24 (32.9%)	19 (30.6%)
Lobar destruction	12 (3.0%)	0	0	3 (4.8%)	4 (5.5%)	2 (3.2%)
≥10% Residual consolidation	31 (7.7%)	3 (27.3%)	1 (1.4%)	3 (4.8%)	12 (16.4%)	5 (8.1%)
≥5% Residual cavitation	21 (5.2%)	1 (9.1%)	2 (2.8%)	3 (4.8%)	7 (9.6%)	3 (4.8%)

*Denominators: Death: all participants recruited to study; Spirometry decline: participants with ATS standard spirometry at TB treatment completion and 1-year visit; Health seeking: participants attending all study visits, or those contributing 6m with ≥1 health seeking episode; Symptoms/limitation & Severe financial impact: participants completing 1-year visit.

^†^Positive TB microbiology: Smear, Xpert TB/Rif, or culture positive at TB diagnosis.

^‡^Clinical questions derived from self-reported answers to St George’s Questionnaire: Regular cough: cough on ≥several days/week, for past 3-months; Regular breathlessness: shortness of breath on ≥several days/week, for past 3-months; Limited walking: needs to walk slower than others, or to stop for a rest; Limitation of activities: chest symptoms preventing 1–2 normal activities, most activities, or all activities.

^¶^Post-bronchodilator spirometry data meeting ATS standards included only (n = 305), and standardised by GLI Black reference ranges.

^CXR readings after dual reporting & consensus review: Ring & tramline markings: moderate to severe markings in ≥1 zone; Lobar destruction: ≥1 lobe, with ≥90% of parenchyma affected by atelectasis / banding / cavities; Residual consolidation: consolidation of ≥10% of parenchyma and Residual cavitation: cavitation of ≥5% of parenchyma (cut offs for both selected as between 90^th^-95^th^ centiles across cohort).

A higher proportion of those experiencing accelerated spirometry decline were female, compared to those with more stable or improving spirometry (40.8% vs. 26.1%, p = 0.017). Baseline spirometry was more likely to be normal at treatment completion, and baseline BMIs higher amongst those with accelerated decline (p = 0.008 and p<0.001 respectively), perhaps reflecting some regression to the mean.

The presence of symptoms including regular cough, regular breathlessness, slower walking pace, or limitation of activities at TB treatment completion were associated with unscheduled respiratory health seeking, symptoms or functional limitation, and self-reported severe financial impact of TB disease by 1-year after treatment completion.

Poverty at TB treatment completion, defined by having a maximum of primary school education or being in the lowest two socioeconomic quintiles in urban Blantyre, was associated with reporting a severe financial impact from TB disease (p = 0.024 and p = 0.005, respectively).

### Predictors of adverse outcomes, in pre-specified generalised logistic regression models

Multivariable regression models constructed to identify predictors of death were unstable (Table A in S4 Text). Across all other models, demographic and clinical parameters were consistently associated with outcomes. Few statistically significant associations were observed between pre-selected spirometry or imaging parameters in adjusted models, when added alongside demographic and clinical variables (Table 3, Tables B-E in S4 Text).

**Table 3:** Adjusted odds ratios estimating associations between pre-specified parameters and outcomes, by logistic regression model, including all pre-specified covariates with no variable reduction.

	Spirometry decline (n = 71/305) OR (95% CI)	Health seeking (n = 62/368) OR (95% CI)	Symptoms / limitation (n = 73/368) OR (95% CI)	Severe financial impact (n = 62/368) OR (95% CI)
Demographic parameters				
Male sex	**0.3 (0.1, 0.7)**	0.9 (0.4, 2)	0.5 (0.2, 1.3)	0.7 (0.3, 1.8)
Age (yrs)	1 (1, 1.1)	**1 (1, 1.1)**	1 (1, 1.1)	**1.1 (1, 1.1)**
Maximum education level > primary school	1.3 (0.6, 2.7)	1.6 (0.7, 3.5)	0.9 (0.4, 2.1)	0.9 (0.4, 1.9)
Positive TB microbiology*	1 (0.4, 2.2)	1.2 (0.6, 2.8)	0.9 (0.4, 2.2)	0.6 (0.3, 1.4)
HIV status (n = 403)				
• Negative	1.	1.0	1.0	1.0
• Positive, CD4 > = 200	0**0.3 (0.1, 0.7)**	**0.5 (0.2, 1)**	0.5 (0.2, 1.1)	1.7 (0.7, 4)
• Positive, CD4<200	0.5 (0.2, 1.1)	0.4 (0.2, 1)	0.6 (0.2, 1.5)	2.5 (1, 6.2)
Ever smoked	1.3 (0.6, 3)	0.5 (0.2, 1.1)	1.5 (0.6, 3.6)	2.2 (1, 5.1)
Main fuel*				
• Charcoal	1.0	1.0	1.0	1.0
• Electricity	3.6 (0.9, 13.8)	0.4 (0, 2)	-	0.7 (0, 4)
• Wood	0.6 (0.2, 1.7)	0.8 (0.3, 2.2)	1.1 (0.4, 3)	0.8 (0.3, 2.2)
Poorest 2 SES quintiles	**2.8 (1.2, 6.6)**	1.6 (0.8, 3.6)	1 (0.4, 2.3)	**2.4 (1.1, 5.2)**
Clinical parameters at TB treatment completion				
BMI (kg/m^2^) median	**1.2 (1, 1.3)**	1 (0.9, 1.1)	0.9 (0.8, 1)	0.9 (0.8, 1.1)
Weekly cough	3.4 (0.1, 47.9)	0.9 (0.1, 7.1)	0.9 (0.1, 7.4)	2.2 (0.2, 18.1)
Weekly breathlessness	**20.5 (3, 199.1)**	**10.2 (2.4, 50)**	**16.7 (3.3, 133.4)**	0.3 (0, 2.3)
Limited walking	0.4 (0.2, 1.1)	0.9 (0.4, 2)	1.1 (0.5, 2.4)	0.6 (0.3, 1.5)
Limitation of activities	2.1 (1, 4.6)	1.8 (0.8, 3.9)	**4.2 (1.8, 10.3)**	**2.3 (1.0, 5.0)**
Spirometry at TB treatment completion				
10% larger FEV_1_% predicted	1.1 (0.7, 1.8)	0.8 (0.5, 1.3)	**0.5 (0.3, 0.9)**	0.9 (0.5, 1.5)
10% larger FVC % predicted	1.7 (1, 2.9)	1.1 (0.6, 1.8)	**1.8 (1, 3.3)**	1.2 (0.7, 2)
Spirometry pattern				
• Normal	1.0	1.0	1.0	1.0
• Obstruction	0.9 (0.2, 3.4)	0.7 (0.2, 2.6)	0.3 (0.1, 1.1)	0.5 (0.1, 2.2)
• Low FVC	1 (0.3, 3.3)	1.3 (0.4, 3.6)	0.7 (0.2, 2.2)	1 (0.3, 3.1)
Imaging at TB treatment completion				
Lobar destruction*	-	1.5 (0.2, 8.5)	0.9 (0.1, 5.6)	2.1 (0.2, 12.7)
Ring & tramline markings	0.8 (0.4, 1.9)	0.8 (0.4, 1.7)	0.8 (0.3, 1.7)	1 (0.4, 2.2)
≥10% Residual consolidation	0.2 (0, 1.2)	0.2 (0, 1)	1.8 (0.5, 5.8)	1.6 (0.4, 5.3)
≥5% Residual cavitation	1.7 (0.2, 11.4)	0.8 (0.1, 3.2)	1.3 (0.3, 5.2)	0.7 (0.1, 3.2)

*Variables where ‘-‘ given are unstable and OR not interpretable.

**Fig 2:**
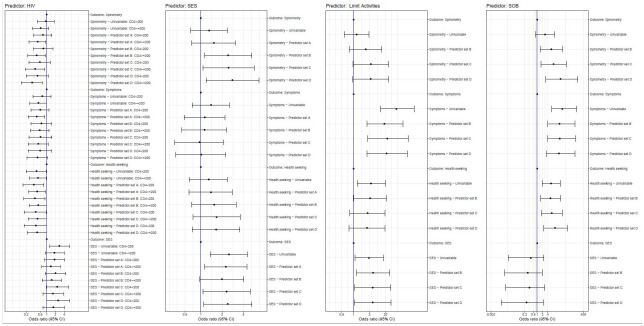
Forest plots describing the associations between key predictors (HIV status, Socioeconomic status, and symptoms) at TB treatment completion, and outcomes at 1-year, in univariable / multivariable models.

Patients with regular breathlessness at TB treatment completion had significantly higher odds of an accelerated spirometry decline, ongoing health seeking, and symptoms or functional limitation at 1-year. These associations were large and statistically significant across models (Fig 2). Those reporting activity limitation at treatment completion had consistently increased odds of respiratory symptoms or activity limitation at 1-year, and were more likely to report a severe financial impact of TB disease (Table 3, Tables D & E in S4 Text). Weekly cough at treatment completion was not correlated with respiratory, health seeking or socioeconomic outcomes.

HIV-positive participants had lower odds of accelerated spirometry decline and unscheduled respiratory health seeking in the year after TB treatment completion compared to HIV-negative participants. This association persisted in models controlling for the extent of PTLD (symptoms, spirometry and imaging) at treatment completion.

Poverty was associated with both adverse socioeconomic outcome and accelerated spirometry decline: in models controlling for the extent of PTLD at TB treatment completion, participants in the poorest two socioeconomic quintiles had 2.4 (95% CI: 1.1–5.2) and 2.8 (95% CI: 1.2–6.6) fold higher odds of these outcomes respectively, compared to those in wealthier quintiles. Males had reduced odds of spirometry decline compared to females across all models, but gender was not associated with any other patient outcome.

Spirometry at TB treatment completion was predictive of symptoms or functional limitation at 1-year only. Although no association was observed with the pattern of spirometry deficit at treatment completion, individuals with higher FEV_1_% predicted values at TB treatment completion had lower odds of symptoms or limitation at 1-year, whilst those with higher FVC % predicted values had increased odds of ongoing symptoms or limitation at 1-year. CXR parameters at treatment completion were not associated with any of the patient outcomes.

A sensitivity analysis investigating the relationship between pre-specified parameters and outcomes amongst those with microbiologically confirmed PTB disease only demonstrated similar patterns of association (S5 Text).

### Predictors of adverse outcomes, with penalised logistic regression models using pre-specified variables

The variable reduced models showed similar relationships to those seen above (S6 Text). Several variables were dropped in the models for respiratory symptoms or limitation at 1-year, but the presence of symptoms at treatment completion remained the most important predictor of outcome: participants with weekly breathlessness or limitation of activities at treatment completion had 16.6 (3.6–122.7) and 4.2 (1.8–10.0) fold higher odds of reporting either of these symptoms one year later, compared to those without symptoms at treatment completion.

HIV was retained as a predictor of spirometry decline or ongoing health seeking. The strength and significance of associations with clinical outcomes was similar, but HIV-status was also significantly associated with socioeconomic outcomes here: HIV-positive adults with CD4 counts <200cells/mm^3^ had 2.5 (1.0–6.2) times higher odds of reporting severe financial effects of TB disease, compared to HIV-negative adults, controlling for the extent of lung pathology (S6 Text).

In the reduced model for spirometry decline, a positive correlation was observed between FVC % predicted at TB treatment completion and accelerated spirometry decline in the following year. However, no other statistically significant associations were observed between spirometry measures or CXR parameters at TB treatment completion, and the various outcomes.

### Predictors of adverse outcomes, with penalised regression models derived from the full parent dataset

The variable importance estimates (S7 Text) and models fit from the full parent dataset (S8 Text) confirm that variables describing participant demographic, socioeconomic, and symptom characteristics at TB treatment completion were the most important predictors of patient outcome. The precise variables included to represent these concepts were different to those pre-selected for use in the models above, but the broad themes were consistent.

Clinical measurements including oxygen saturation, heart rate, and 6-minute walk distance at TB treatment completion also emerged as important predictors of patient outcomes using this approach. However, the importance of spirometry and CXR parameters remained limited, with statistically significant associations observed between FVC % predicted and % parenchymal abnormality, and spirometry decline only.

### Model performance

**Fig 3:**
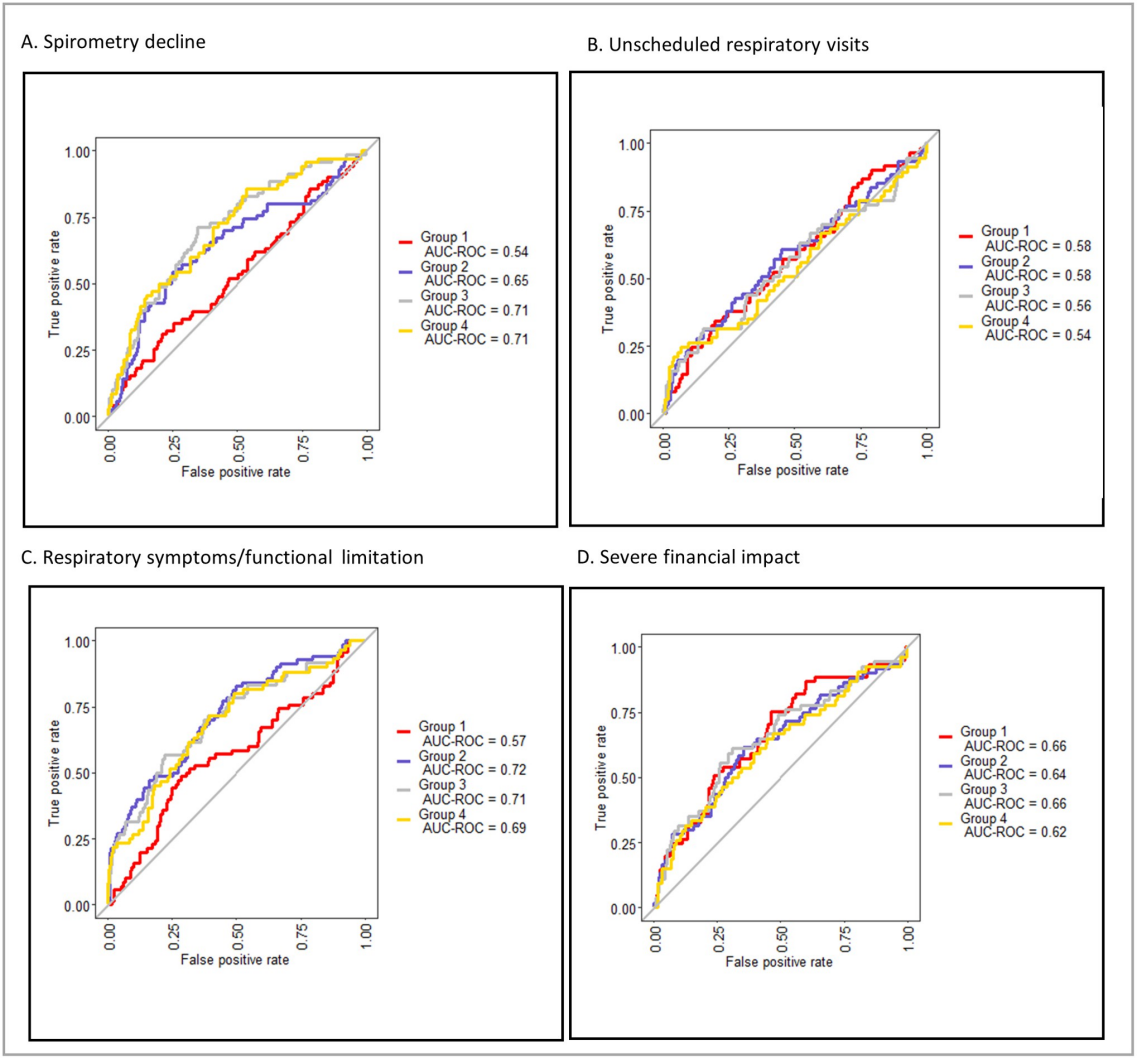
ROC curves for logistic regression models for each outcome, using pre-specified predictor variable sets with no variable reduction. Group 1: Demographic variables only; Group 2: Demographic plus clinical variables; Group 3: Demographic, clinical, and spirometry variables; Group 4: Demographic, clinical, spirometry and CXR variables.

**Fig 4:**
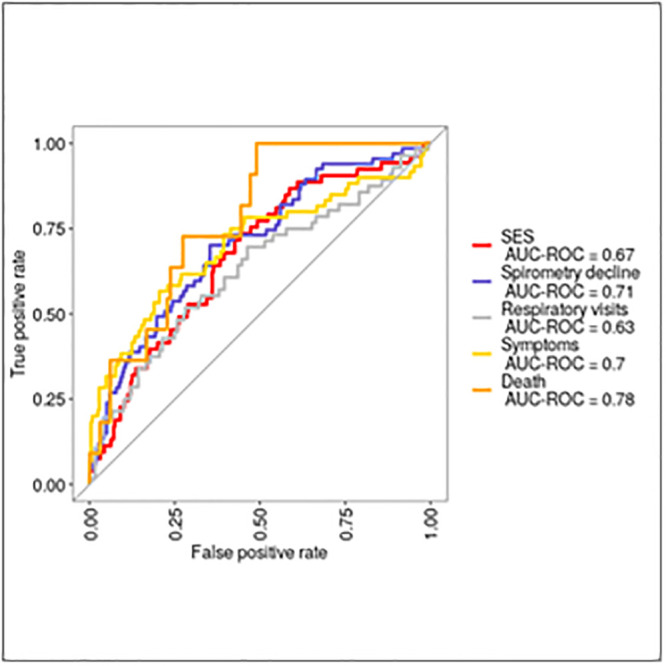
ROC curves for logistic regression models for each outcome, using penalised regression of full parent data set.

Regardless of the predictor set or variable selection process used, the ROC curves generated for all outcomes showed limited AUC, suggesting limited predictive power. There was little difference between the AUCs generated by the full and penalised regression models constructed using the pre-selected variable sets (Fig 3, S9 Text), and those derived from the full parent data set (Fig 4), suggesting that the *a priori* predictor sets captured the main constructs of value.

Death was the hardest variable to predict with unstable estimation due to small numbers. The AUCs observed for models of health seeking and the financial impact of TB disease were limited (maximum 0.65 and 0.66), whilst those for the models of symptoms and spirometry decline were higher (maximum 0.77 and 0.74). The largest increments were observed when clinical and symptom parameters were added to baseline demographic and socioeconomic characteristics in the models constructed using the a-priori data sets. Little further improvement in AUC was seen when spirometry and CXR parameters were added (Fig 3).

### Performance of individual symptom questions in screening for adverse outcomes

Because symptoms at TB treatment completion were highlighted across analyses as key predictors, the sensitivity, specificity, positive and negative predictive values (PPV and NPV) of a range of individual symptom questions for outcomes were examined. Specificity and PPVs were limited, but sensitivity and NPVs were higher (S10 Text). The performance of individual symptom questions was superior to binary spirometry and imaging parameters (S11 Text).

The symptom questions with the highest NPV across outcomes were limitation of activities (reported by 205/403 (50.6%), NPV 78.2–98.2%), and breathlessness when walking up hills (reported by 176/405 (43.5%), NPV 77.4–99.5%), with the highest values seen for mortality and ongoing symptoms or limitation at 1-year (Table 4). Similar patterns were observed in a sensitivity analysis of those treated for microbiologically confirmed disease only (S12 Text).

**table 4:** Performance of question about limitation of activity and shortness of breath on hills at TB treatment completion, in screening for adverse patient outcomes.

Symptom question at TB treatment end	Outcome, in the year after treatment completion	Sensitivity	Specificity	PPV	NPV
Limitation of activities (n = 205/403, 50.6%)	Death	72.7%	48.9%	4.5%	98.2%
	Spirometry decline	53.7%	50.9%	25.2%	78.2%
	Respiratory health seeking	66.1%	51.4%	23.1%	87.2%
	Symptoms or activity limitation	80.0%	55.8%	30.4%	92.1%
	Severe financial impact	64.2%	51.6%	21.5%	87.4%
Breathlessness on hills (n = 176/405, 43.5%)	Death	90.9%	57.1%	6.6%	99.5%
	Spirometry decline	43.3%	59.6%	24.8%	77.4%
	Respiratory health seeking	64.3%	62.1%	27.3%	88.7%
	Symptoms or activity limitation	68.3%	64.3%	31.5%	89.4%
	Severe financial impact	52.8%	60.2%	21.5%	86.0%

The prevalence of abnormal patterns of spirometry, residual cavitation, and consolidation on imaging were significantly higher amongst those who reported limitation of activities at treatment completion, compared to those who did not (S13 Text).

## Discussion

In this secondary analysis of prospective cohort data from Malawi, we show that it is challenging to accurately predict morbidity after TB treatment completion at the individual level, but that demographic, clinical, and symptom parameters are strongly associated with adverse outcomes. Simple symptom questions at treatment completion have moderate negative predictive value for morbidity outcomes within this data set, with little incremental benefit from the additional use of spirometry and imaging.

We have previously shown that a substantial minority of adults completing treatment for PTB experience adverse respiratory, health seeking, and socioeconomic outcomes in the year after TB treatment completion [6,9]. The further analyses conducted here show that it is difficult to predict these outcomes at the individual level: ROC AUC values for all of the predictive models developed were poor, and changed little with the different modelling approaches used, suggesting that this is not a function of the variables included but rather a genuine limitation of outcome prediction within this dataset. However, demographic factors and symptoms were strong predictors of morbidity across these models, with individual symptom questions showing moderate NPVs for adverse outcomes. This was in contrast to imaging and spirometry parameters, which were poorly associated with patient outcomes and did little to improve the predictive capacity of models.

Our findings suggest that it will likely be challenging to develop a screening tool which accurately predicts adverse outcomes amongst PTB survivors at the individual level. One possible response to this finding–if confirmed in other data sets–would be to offer routine post-TB follow up to all PTB survivors, accepting that only a small proportion of these individuals will go on to develop post-TB morbidity. This would be an inclusive approach and may provide reassurance to many patients. However, significant funding and staff resources would be required to implement this approach. There may also be patient harms associated with routine follow-up including the ongoing direct and indirect costs of additional health care visits [18], stigma related to ongoing contact with TB services [19], and anxiety around a diagnosis of chronic condition, and uptake may be limited amongst those who are asymptomatic at treatment completion. In-depth qualitative work and health economic analyses would be needed to explore the patient and health system implications of routine post-TB follow up, and likely demand for these services.

Where there is a wish to provide more targeted care to a limited number of TB survivors only, our data suggest that a symptom screening approach could be used to direct this. The absence of symptoms such as exertional breathlessness and functional impairment at TB treatment completion could be used as a ‘rule out’ test, with those who are asymptomatic discharged from care. We acknowledge that there are limitations with this approach. Symptoms are a relatively blunt tool and within our data set still only half of patients would be asymptomatic and discharged at TB treatment completion using this approach. Between 13–22% of those who are discharged may go on to experience accelerated spirometry decline or respiratory health seeking, or report a severe financial impact of TB disease. Meanwhile, amongst those symptomatic patients who receive ongoing follow-up, still only a minority would go on to develop adverse outcomes.

Although this is also an imperfect approach, it is low cost, requires minimal training and time, could be implemented at the programmatic level, and would half the number of patients offered follow-up after treatment completion. Post-TB services built around patient symptoms may be more acceptable and readily adopted by patients who still feel unwell and perceive a need for ongoing review [20], and points of re-entry in to respiratory or post-TB care could be established for those who are discharged but deteriorate over time [21]. A symptom based approach may also allow those with other causes of chronic symptoms to be identified, including non-TB chronic respiratory diseases (E.g. COPD, occupational lung diseases), cardiovascular disease, deconditioning and muscle loss related to severe illness, anaemia, or advanced HIV, with linkage of symptomatic patients to ongoing care [22,23]. There may therefore be several positive outcomes from such an approach.

Of note–our data suggest that the ability to predict adverse outcomes does not improve with the addition of spirometry and CXR data to simple symptom screening. Where NTPs are interested in focused post-TB care, it may therefore be reasonable to use simple symptom questionnaires to direct this, without further investigation. This finding is particularly relevant to low-resource settings, where care is decentralised and delivered in health centres by nursing staff and TB officers with limited access to respiratory diagnostics. The choice of symptom questions and their wording will require optimisation, and may vary between settings.

Lastly, in this analyses we have investigated the use of screening approaches to identify those at risk of five types of post-TB morbidity. However, as we develop clinical interventions for post-TB patient care, screening approaches may need to be modified in order to identify patients who are eligible for these interventions, and the use of imaging and spirometry alongside symptoms may need to be reconsidered. For example, studies of pulmonary rehabilitation for PTB survivors in LMICs currently use breathlessness to determine eligibility, in an approach similar to that described here, with good preliminary results [24,25]. However, interventions such as inhaled bronchodilators are most effective in those with airway obstruction, and spirometry may be needed at the point of TB treatment completion to identify individuals those with obstructive airways disease who are eligible for inhaled therapies.

This study has several important limitations. It was an exploratory secondary analysis, and might therefore be subject to bias. We constructed models for outcomes chosen by the study team, which have not been recognised or validated outside of this study, and we did not consider eligibility for clinical interventions. We were unable to develop strong predictive models for death in the year after TB treatment completion–this was likely due to low event numbers, but may also reflect true challenges in predicting this outcome, and larger data sets will be needed to explore this. The data used were collected from a single study, with a single approach to measuring symptoms, imaging and spirometry, in a single site. The PPV and NPV reported for predicted may differ in other sites where the incidence of adverse outcomes in the post-TB period differs. Critically, the patient outcomes evaluated were measured at 1-year after treatment completion only. We also explored parallel use of tests only, and did not explore a two- or three-stage screening process with sequential use of tests. Although we hypothesise that a symptom-based approach would be cost-effective and acceptable, this was not formally evaluated. These issues must be explored in other datasets and settings.

However, despite these limitations, our analysis is relevant to the development of post-TB care services in LMICs, and provides evidence to inform NTP decision making about if and how to screen patients at TB treatment completion, in order to determine access to post-TB care. We show that it is challenging to accurately identify those at greatest risk of adverse post-TB outcomes at TB treatment completion. However, if there is a need to prioritise ongoing care, the use of low cost symptom based screening tools may be the most promising approach with little incremental benefit from the additional use of imaging or spirometry. Symptom questions must be refined and optimised, and this symptom-based approach may need to evolve as targeted post-TB interventions become more widely available. However, this is a pragmatic approach which could be used by NTPs at TB treatment completion, as they seek to monitor and improve the long-term wellbeing of TB survivors.

## Supporting information

S1 Text Scoring database for CXR reporting. (DOCX)

S2 Text Parameters measured at TB-treatment completion, and pre-selected for inclusion in predictive models for adverse outcomes in the subsequent year. (DOCX)

S3 Text Full set of parameters measured at TB-treatment completion within parent study, and included in elastic net penalised regression models for adverse patient outcomes. (DOCX)

S4 Text **Table A:** Univariate and multivariable associations between pre-specified parameters and death, in logistic regression models, with no variable reduction but with Firth’s correction applied (n = 405). **Table B:** Univariate and multivariable associations between pre-specified parameters and accelerated spirometry decline, in logistic regression models, with no variable reduction (n = 305). **Table C:** Univariate and multivariable associations between pre-specified parameters and unscheduled respiratory health seeking, in logistic regression models, with no variable reduction (n = 368). **Table D:** Univariate and multivariable associations between pre-specified parameters and chronic respiratory symptoms, in logistic regression models, with no variable reduction (n = 368). **Table E:** Univariate and multivariable associations between pre-specified parameters and self-reported severe financial impact of disease, in logistic regression models, with no variable reduction(n = 368).
(DOCX)

S5 Text Adjusted odds ratios estimating associations between pre-specified parameters and outcomes, by logistic regression model, including all pre-specified covariates with no variable reduction in microbiology confirmed TB participants only. (DOCX)

S6 Text Multivariable associations (OR (95% CI) between pre-specified parameters and outcomes, in penalised regression models. (DOCX)

S7 Text Variable importance values for prediction of outcome, using full parent data set (Higher value indicates greater importance in predicting outcome). (DOCX)

S8 Text Models constructed for each outcome, from full parent data set. (DOCX)

S9 Text ROC curves for logistic regression models for each outcome, using pre-specified predictor variable sets after variable reduction with penalised regression (Group 1: Demographic only; Group 2: Demographic plus clinical; Group 3: Demographic, clinical, spirometry; Group 4: Demographic, clinical, spirometry and CXR). (DOCX)

S10 Text Sensitivity, specificity, and predictive values of individual symptoms questions for each outcome. (DOCX)

S11 Text Performance of indicators on CXR at TB treatment completion, in screening for adverse patient outcomes. (DOCX)

S12 Text Performance of question about limitation of activity and shortness of breath on hills at TB treatment completion, in screening for adverse patient outcomes among TB microbiology confirmed participants only. (DOCX)

S13 Text Patient characteristics, stratified by presence or absence of limitation of activities at TB treatment completion. (DOCX)
