# Supplementary material for: Screening for post-TB lung disease at TB treatment completion: Are symptoms sufficient?
Source: PLOS Glob Public Health. 2024 Jan 29;4(1):e0002659. doi: 10.1371/journal.pgph.0002659 (PMC10824425; doi:10.1371/journal.pgph.0002659)
Supplement: S1 Text — (DOCX) [file pgph.0002659.s001.docx]

# Supplementary materials 1: Chest X-Ray analysis procedures

A dedicated CXR reporting tool was developed by the study team to capture parenchymal, airways, pleural and mediastinal pathology felt likely to be common after TB treatment completion, based on previous literature search (Table 1).

Each CXR was split into 6 lung zones for reporting, with areas delineated using 2 continuous horizontal lines drawn across the CXR image, at approximately the level of the aortic knuckle and lower edge of the wide section of the pulmonary artery, with the aim of dividing each lung field in to 3 ~equally sized sections. Parenchymal and airway features were reported by zones, whilst pleural and mediastinal features were reported at the level of the hemithorax/overall image.

Table A: Scoring database for CXR reporting

| **Lung Zone** | | **R upper** | **L upper** | **R middle** | **L middle** | **R lower** | **L lower** |
| --- | --- | --- | --- | --- | --- | --- | --- |
| **Parenchymal pathology (to nearest 5%, summing to 100%)** | Normal |  |  |  |  |  |  |
|  | Parenchymal bands |  |  |  |  |  |  |
|  | Consolidation |  |  |  |  |  |  |
|  | Ground glass opacities |  |  |  |  |  |  |
|  | Emphysematoid destruction |  |  |  |  |  |  |
|  | Cavities |  |  |  |  |  |  |
|  | Atelectasis |  |  |  |  |  |  |
| **Additional features** | Nodules (0-3)* |  |  |  |  |  |  |
|  | Ring / tramline opacities (0 - 3)* |  |  |  |  |  |  |
| **Cavities** | Number |  |  |  |  |  |  |
|  | Size of largest (2 dimensions) |  |  |  |  |  |  |
|  | Mycetoma (Y/N) |  |  |  |  |  |  |

*0-3 corresponding to nil, mild, moderate, severe

| **Hemithorax** | **Right** | **Left** |  |  |  |  |  |  |  |  |  |  |
| --- | --- | --- | --- | --- | --- | --- | --- | --- | --- | --- | --- | --- |
| Hyperexpansion (Y/N) |  |  |  |  |  |  |  |  |  |  |  |  |
| Pleural effusion (Y/N) |  |  |  |  |  |  |  |  |  |  |  |  |
| Pleural thickening (Y/N) |  |  |  |  |  |  |  |  |  |  |  |  |
| Lymph nodes (Y/N) |  | |  |  |  |  |  |  |  |  |  |  |
| Dominant pathology |  | |  |  |  |  |  |  |  |  |  |  |
| Free text |  | |  |  |  |  |  |  |  |  |  |  |

Digital CXRs were independently reported by two readers:

- JM – Respiratory registrar
- EJ – Consultant radiologist, with specialist interest in tuberculosis

Consensus review was completed by discussion between readers, in order to generate final scores
