# Supplementary material for: Screening for post-TB lung disease at TB treatment completion: Are symptoms sufficient?
Source: PLOS Glob Public Health. 2024 Jan 29;4(1):e0002659. doi: 10.1371/journal.pgph.0002659 (PMC10824425; doi:10.1371/journal.pgph.0002659)
Supplement: S2 Text — (DOCX) [file pgph.0002659.s002.docx]

S2 Table: Parameters measured at TB-treatment completion, and pre-selected for inclusion in predictive models for adverse outcomes in the subsequent year

| Category | Group | Variable description |
| --- | --- | --- |
| Demographic data | Age | Participant age at TB treatment completion |
|  | Gender | Participant gender |
|  | Education level | Education beyond primary school (Primary & nil vs. secondary and above) |
|  | SES | Richest 3 vs. poorest 2 urban wealth quintiles, derived from the |
|  | Microbiology proven pTB | Smear, culture or GXP positive at TB diagnosis |
|  | HIV status and CD4 count | Composite categorical variable, denoting HIV negative, HIV positive wit CD4 <200, and HIV positive with CD4 ≥200 at TB treatment completion |
|  | Ever smoked | Binary variable |
|  | Main fuel | Type of fuel mainly used by the household for cooking |
| Clinical variables | Regular cough | cough ≥ few days per month for past 3m |
|  | Regular SOB | SOB ≥ few days per month for past 3m |
|  | Limitation of general activity | Chest stops me doing 1-2 things, most of the things I would like, or everything |
|  | Limited walking pace | Walk slower than others / stop for rests |
|  | BMI | BMI (kg/m3) at TB treatment completion |
| Spirometry | FEV 10% predicted | Post bronchodilator FEV1 10% predicted values, using GLI reference ranges |
|  | FVC 10% predicted | Post bronchodilator FVC 10% predicted values, using GLI reference ranges |
|  | Pattern of spirometry deficit | Obstruction (FEV/FVC ratio<LLN), low FVC (FEV/FVC ratio>LLN & FVC<LLN), or normal spirometry |
|  | Any abnormal spirometry | Either of obstruction or low FVC pattern, grouped together |
| CXR variables | Parenchymal pathology - specific patterns | ≥10% Residual consolidation, across whole lung |
|  |  | ≥5% Residual cavitation, across whole lung |
|  | Parenchymal pathology - combining patterns | At least 1 lobe with ≥90% absent parenchyma due to cavities/atelectasis/banding |
|  | Bronchiectasis variables | Any ring & tramlines, at least moderate |
