# Supplementary material for: Screening for post-TB lung disease at TB treatment completion: Are symptoms sufficient?
Source: PLOS Glob Public Health. 2024 Jan 29;4(1):e0002659. doi: 10.1371/journal.pgph.0002659 (PMC10824425; doi:10.1371/journal.pgph.0002659)
Supplement: S3 Text — (DOCX) [file pgph.0002659.s003.docx]

S3 Table: Full set of parameters measured at TB-treatment completion within parent study, and included in elastic net penalised regression models for adverse patient outcomes

| Category | Group | Variable description |
| --- | --- | --- |
| Demographic data | Age * | Participant age at TB treatment completion |
|  | Gender * | Participant gender |
|  | Education level * | Education beyond primary school (Primary & nil vs. secondary and above) |
|  | SES * | Richest 3 vs. poorest 2 urban wealth quintiles, derived from the…… |
|  | Food insecurity | Difficulty procuring food for the household experienced sometimes/often |
|  | Missing meals | Adult in the household skipped a meal at least once in the past 2 weeks, to feed children |
|  | Financial impact of disease | Self reported financial impact of disease, by point of treatment completion |
|  | Dissaving during disease & treatment | Household incurred dissaving (use of savings, borrowing money, selling assets) during the year prior to treatment completion, to cover costs |
|  | Interruption of schooling | Child in household pulled out of education during previous year |
|  | Microbiology proven pTB * | Smear, culture or GXP positive at TB diagnosis |
|  | Illness duration | Self reported illness duration (wks) prior to diagnosis |
|  | HIV status and CD4 count * | Composite categorical variable, denoting HIV negative, HIV positive wit CD4 <200, and HIV positive with CD4 ≥200 at TB treatment completion |
|  | Ever smoked * | Binary variable |
|  | Main fuel * | Type of fuel mainly used by the household for cooking |
| Clinical observations | BMI | BMI (kg/m3) at Tb treatment end |
|  | Heart rate | Heart rate (BPM) at rest |
|  | Tachycardia | Tachycardia >100bpm at rest |
|  | Saturations | Oxygen saturation at rest |
|  | Hypoxia | Hypoxia (sats<96%) at rest |
|  | Respiratory rate | Respiratory rate at rest (resps per minute) |
|  | Tachypnoea | Tachypnoea (RR>20) at rest |
|  | 6MWD | Distance walked in 6-minute walking test |
| Clinical variables | SGRQ symptom score | SGRQ Symptoms score |
|  | SGRQ Activity score | SGRQ Activity score |
|  | SGRQ impact score | SGRQ Impact score |
|  | SGRQ total score | SGRQ Total score |
|  | Regular cough * | cough ≥ few days per month for past 3m |
|  | Regular SOB * | SOB ≥ few days per month for past 3m |
|  | Regular sputum production | Sputum production ≥ few days per month for past 3m |
|  | Regular wheeze | Wheeze ≥few days per month for past 3m |
|  | Any monthly respiratory symptoms | Any resp symptom ≥few days/month in past 3m (cough,sputum,sob,wheeze) |
|  | Any weekly respiratory symptoms | Regular resp symptom in past 3m, ≥several days/week (cough, sputum, sob, wheeze |
|  | Chest attacks | At least one severe / unpleasant attack of chest trouble in past 3m |
|  | Good days | Good days in average week, over past 3m |
|  | Problems relating to chest | Chest problem causes quite a lot of problems, or is the most important problem |
|  | Effect of chest trouble on work | Chest trouble interferes with / made me stop work |
|  | Limitation of general activity * | Chest stops me doing 1-2 things, most of the things I would like, or everything |
|  | SOB at rest | Usually SOB when sitting/lying still |
|  | SOB during self care | Usually SOB when getting washed/dressed |
|  | SOB walking at home | Usually SOB when walking around home |
|  | SOB walking outside | Usually SOB when walking outside on flat |
|  | SOB on incline | Usually SOB when walking up hills |
|  | Slow on washing/dressing | Take a long time to get washed/dressed |
|  | Slow on bathing/showering | Cannot take bath/shower, or take a long time |
|  | Limited walking pace * | Walk slower than others / stop for rests |
|  | Slow on housework | Jobs like housework take a long time / stop for rests |
|  | Slow on walking up stairs | Goes slow / stop when walking up 1 flight of stairs |
|  | Slow on hurrying | Struggles to hurry / walk fast |
|  | Severe SOB | Breathing makes light activities difficult - walking up hills, carrying things up stairs, light garden, dancing |
|  | Moderate SOB | Breathing makes moderate activities difficult – carrying heavy loads, digging the garden, walking fast, jogging |
|  | Mild SOB | Breathing makes very strenuous activities difficult - heavy manual work, running, cycling |
|  | Exhaustion | Gets exhausted quickly |
|  | Self reported frailty | Feels has become frail / an invalid because of my chest |
|  | Difficulty farming | Self reported difficulty farming due to chest trouble |
|  | Difficulty lifting things | Self reported difficulty lifting heavy items due to chest trouble |
|  | Difficulty with manual work | Self reported difficulty with heavy duties/manual work due to chest trouble |
|  | EQ5D3L mobility score | EQ5D3L mobility |
|  | EQ5D3L self care score | EQ5D3L self-care |
|  | EQ5D3L activity score | EQ5D3L usual activities |
|  | EQ5D3L pain score | EQ5D3L pain/discomfort |
|  | EQ5D3L activity / depression score | EQ5D3L activity/depression |
|  | EQ5D3L Visual analogue score | Visual Analogue Score |
|  | 6-minute walking distance | Distance walked (m) in 6-minute walk test |
| Spirometry | FEV 10% predicted * | Post bronchodilator FEV1 10% predicted values, using GLI reference ranges |
|  | FVC 10% predicted * | Post bronchodilator FVC 10% predicted values, using GLI reference ranges |
|  | FEV/FVC ratio | Post-bronchodilator FEV/ FVC ratio |
|  | Pattern of spirometry deficit * | Obstruction (FEV/FVC ratio<LLN), low FVC (FEV/FVC ratio>LLN & FVC<LLN), or normal spirometry |
|  | Any abnormal spirometry * | Either of obstruction or low FVC pattern, grouped together |
|  | Airway obstruction | Any airway obstruction (Ratio<LLN GLI), vs. normal spirometry |
|  | Low FVC pattern | LowFVC (Ratio>LLN GLI & FVC<LLN GLI), vs. normal spirometry |
| CXR variables | Parenchymal pathology - specific patterns | % Atelectasis, across whole lung |
|  |  | % consolidation, across whole lung * |
|  |  | % parenchymal banding, across whole lung |
|  |  | % cavities, across whole lung * |
|  | Parenchymal pathology - combining patterns | % of abnormal parenchyma of any pattern, across whole lung |
|  |  | Number of lobes with ≥90% absent parenchyma due to cavities/atelectasis/banding |
|  |  | At least 1 lobe with ≥90% absent parenchyma due to cavities/atelectasis/banding * |
|  | Bronchiectasis variables | Total lung ring & tramline score |
|  |  | Number of lobes with ring & tramlines, at least moderate |
|  |  | Any ring & tramlines, at least moderate * |
|  | Nodules | Number of lobes with nodules |
|  |  | Any nodules |
|  |  | Presence of any miliary pattern in lung |
|  | Whole-lung findings | Any mycetoma |
|  |  | Presence of any hyperexpansion |
|  |  | Presence of pleural pathology - effusions or thickening, L or R |

*Variable included in apriori set of predictors also
