# Supplementary material for: Screening for post-TB lung disease at TB treatment completion: Are symptoms sufficient?
Source: PLOS Glob Public Health. 2024 Jan 29;4(1):e0002659. doi: 10.1371/journal.pgph.0002659 (PMC10824425; doi:10.1371/journal.pgph.0002659)
Supplement: S4 Text — Table A: Univariate and multivariable associations between pre-specified parameters and death, in logistic regression models, with no variable reduction but with Firth’s correction applied (n = 405). Table B: Univariate and multivariable associations between pre-specified parameters and accelerated spirometry decline, in logistic regression models, with no variable reduction (n = 305). Table C: Univariate and multivariable associations between pre-specified parameters and unscheduled respiratory health seeking, in logistic regression models, with no variable reduction (n = 368). Table D: Univariate and multivariable associations between pre-specified parameters and chronic respiratory symptoms, in logistic regression models, with no variable reduction (n = 368). Table E: Univariate and multivariable associations between pre-specified parameters and self-reported severe financial impact of disease, in logistic regression models, with no variable reduction(n = 368). (DOCX) [file pgph.0002659.s004.docx]

# Supplementary materials 4: Relationship between pre-specified predictor variables and patient outcomes, in logistic regression models, including all pre-specified predictors

Table A: Univariate and multivariable associations between pre-specified parameters and death, in logistic regression models, with no variable reduction but with Firth’s correction applied (n=405)

| **Outcome: Death** | **Univariate**  **OR (95% CI)** | **Demographic variables**  **OR (95% CI)** | **+ Clinical variables**  **OR (95% CI)** | **+ Spirometry variables**  **OR (95% CI)** | **+ CXR variables**  **OR (95% CI)** |
| --- | --- | --- | --- | --- | --- |
| Male sex | 1.4 (0.2, 28.6) | 1.3 (0.8, 2.2) | 1.1 (0.6, 2.2) | 1.1 (0.4, 2.9) | 1.1 (0.6, 1.8) |
| Age (yrs) | 1.7 (0.9, 1.8) | 1.0 (1.0, 1.0) | 1.0 (1.0, 1.0) | 0.9 (0.9, 1.0) | 1.0 (1.0, 1.0) |
| Maximum education level > primary school | - | 2.1 (1.5, 3.4) | 1.9 (1.2, 3.5) | 1.3 (0.4, 3.9) | 1.0 (0.9, 1.1) |
| Positive TB microbiology* | - | 0.8 (0.4, 2.2) | 0.8 (0.4, 1.4) | 0.8 (0.3, 2.4) | 1.0 (0.6, 1.6) |
| HIV status (n=403) - Negative | 1.0 | 1.0 | 1.0 | 1.0 | 1.0 |
| - Positive, CD4 ≥200 | 0.77 (0.6, 1.0) | 0.7 (0.5, 1.1) | 0.8 (0.4, 1.3) | 1.1 (0.5, 2.6) | 1.0 (0.8, 2.1) |
| - Positive, CD4<200 | - | 4.8 (2.7, 26.6) | 4.0 (1.9, 35.2) | 2.9 (0.9, 24.4) | 1.1 (0.9, 2.4) |
| Ever smoked | 0.02 (0, 237.7) | 0.6 (0.3, 0.9) | 0.5 (0.2, 0.8) | 0.7 (0.3, 1.9) | 1.0 (0.7, 1.7) |
| Main fuel - Charcoal | 1.0 | 1.0 | 1.0 | 1.0 | 1.0 |
| - Electricity | - | - | - | - | 1.1 (0.4, 3.1) |
| - Wood | 1.59 (1.3, 2.0) | 1.6 (0.8, 3.8) | 1.4 (0.6, 3.8) | 1.3 (0.4, 6.7) | 1.1 (0.6, 2.0) |
| Poorest 2 SES quintiles | 0.15 (0, 825.2) | 1.4 (0.9, 2.3) | 1.4 (0.8, 2.6) | 1.1 (0.4, 3.0) | 1.0 (0.9, 1.1) |
| Weekly cough | 0.009 (0, 43.9) |  | 0.6 (0.2, 1.8) | 0.6 (0.1, 6.1) | 1.3 (0.4, 4.1) |
| Weekly breathlessness | 0.02 (0, 69.7) |  | 0.5 (0.2, 1.3) | 0.4 (0.1, 2.6) | 0.9 (0.4, 1.9) |
| Limited walking pace | - |  | 3.0 (1.8, 7.1) | 2.6 (0.8, 10.8) | 1.1 (0.7, 1.7) |
| Limitation of activities | - |  | 0.4 (0.2, 0.6) | 0.5 (0.2, 1.6) | 1.0 (0.9, 1.1) |
| BMI (kg/m^2^) median | 0.7 (0.6, 1.5) |  | 0.9 (0.8, 0.9) | 0.8 (0.7, 1.0) | 1.0 (0.9, 1.1) |
| FEV_1_ 10% predicted | 0.68 (0.3, 4.8) |  |  | 1.0 (1.0, 1.0) | 1.0 (1.0, 1.0) |
| FVC 10% predicted | 1.4 (0.5, 7.9) |  |  | 1.0 (1.0, 1.0) | 1.0 (1.0, 1.0) |
| Pattern  - Normal | 1.0 |  |  | 1.0 | 1.0 |
| - Obstruction | 0.02 (0, 72.4) |  |  | 0.7 (0.2, 2.1) | 0.9 (0.5, 1.6) |
| - Low FVC | 0.02 (0, 16.6) |  |  | 0.6 (0.2, 1.7) | 1.0 (0.5, 1.7) |
| Lobar destruction | 0.01 (0, 53.5) |  |  |  | 0.8 (0.4, 2.5) |
| Ring & tramline markings | 0.02 (0, 237.7) |  |  |  | 1.0 (0.9, 1.1) |
| ≥10% Residual consolidation | 0.02 (0, 111.4) |  |  |  | 1.1 (0.8, 1.9) |
| ≥5% Residual cavitation | 0.01 (0, 79.1) |  |  |  | 1.0 (0.6, 1.8) |

Cells with “-“ indicate that parameter was not estimable

Table B: Univariate and multivariable associations between pre-specified parameters and accelerated spirometry decline, in logistic regression models, with no variable reduction (n=305)

| **Outcome: Spirometry decline** | **Univariate**  **OR (95% CI)** | **Demographic variables**  **OR (95% CI)** | **+ Clinical variables**  **OR (95% CI)** | **+ Spirometry variables**  **OR (95% CI)** | **+ CXR variables**  **OR (95% CI)** |
| --- | --- | --- | --- | --- | --- |
| Male sex | **0.5 (0.3, 0.9)** | **0.4 (0.2, 0.8)** | **0.4 (0.2, 0.9)** | **0.3 (0.1, 0.6)** | **0.3 (0.1, 0.7)** |
| Age (yrs) | 1 (1, 1) | **1 (1, 1.1)** | 1 (1, 1.1) | 1 (1, 1.1) | 1 (1, 1.1) |
| Maximum education level > primary school | 0.8 (0.5, 1.4) | 1.1 (0.6, 2.1) | 1.2 (0.6, 2.5) | 1.2 (0.6, 2.5) | 1.3 (0.6, 2.7) |
| Positive TB microbiology* | 1 (0.5, 1.9) | 1.1 (0.5, 2.2) | 1.2 (0.6, 2.5) | 1 (0.5, 2.3) | 1 (0.4, 2.2) |
| HIV status (n=403) - Negative | 1.0 | 1.0 | 1.0 | 1.0 | 1.0 |
| - Positive, CD4 ≥200 | 0.6 (0.3, 1.2) | **0.5 (0.3, 0.9)** | **0.5 (0.2, 0.9)** | **0.4 (0.2, 0.9)** | **0.3 (0.1, 0.7)** |
| - Positive, CD4<200 | 0.9 (0.5, 1.8) | 0.7 (0.4, 1.5) | 0.8 (0.4, 1.6) | 0.6 (0.2, 1.3) | 0.5 (0.2, 1.1) |
| Ever smoked | 0.8 (0.4, 1.5) | 1.1 (0.5, 2.3) | 1 (0.5, 2.2) | 1.2 (0.5, 2.7) | 1.3 (0.6, 3) |
| Main fuel - Charcoal | 1.0 | 1.0 | 1.0 | 1.0 | 1.0 |
| - Electricity | 1.7 (0.5, 5) | 2.2 (0.6, 6.9) | 2.3 (0.6, 7.6) | 3.9 (1, 14.7) | 3.6 (0.9, 13.8) |
| - Wood | 1.3 (0.5, 2.8) | 0.9 (0.4, 2.2) | 0.9 (0.3, 2.2) | 0.8 (0.2, 2.1) | 0.6 (0.2, 1.7) |
| Poorest 2 SES quintiles | 1.3 (0.7, 2.4) | 1.5 (0.8, 3.1) | **2.4 (1.1, 5.2)** | **2.5 (1.1, 5.6)** | **2.8 (1.2, 6.6)** |
| Weekly cough | 0.8 (0, 5.5) |  | 0.5 (0, 4.8) | 1.2 (0, 15) | 3.4 (0.1, 47.9) |
| Weekly breathlessness | 2.8 (0.8, 9.6) |  | **6.3 (1.5, 27)** | **8.4 (1.7, 44.1)** | **20.5 (3, 199.1)** |
| Limited walking pace | 0.8 (0.4, 1.5) |  | 0.4 (0.2, 1) | 0.5 (0.2, 1.2) | 0.4 (0.2, 1.1) |
| Limitation of activities | 1.1 (0.7, 2) |  | 1.7 (0.8, 3.4) | 2.1 (1, 4.5) | 2.1 (1, 4.6) |
| BMI (kg/m^2^) median | **1.2 (1.1, 1.3)** |  | **1.2 (1.1, 1.4)** | **1.2 (1, 1.3)** | **1.2 (1, 1.3)** |
| FEV_1_ 10% predicted | **1.5 (1.3, 1.8)** |  |  | 1.1 (0.7, 1.8) | 1 (0.6, 1.7) |
| FVC 10% predicted | **1.7 (1.4, 2.1)** |  |  | 1.7 (1, 2.9) | 1.7 (1, 3.1) |
| Pattern  - Normal | 1.0 |  |  | 1.0 | 1.0 |
| - Obstruction | 0.7 (0.3, 1.5) |  |  | 0.9 (0.2, 3.4) | 0.6 (0.1, 2.4) |
| - Low FVC | **0.2 (0.1, 0.6)** |  |  | 1 (0.3, 3.3) | 0.8 (0.2, 2.7) |
| Lobar destruction | - |  |  |  | - - |
| Ring & tramline markings | 0.6 (0.3, 1) |  |  |  | 0.8 (0.4, 1.9) |
| ≥10% Residual consolidation | 0.1 (0, 0.7) |  |  |  | 0.2 (0, 1.2) |
| ≥5% Residual cavitation | 0.5 (0.1, 2) |  |  |  | 1.7 (0.2, 11.4) |

Cells with “-“ indicate that parameter was not estimable

Table C: Univariate and multivariable associations between pre-specified parameters and unscheduled respiratory health seeking, in logistic regression models, with no variable reduction (n=368)

| **Outcome: Health seeking** | **Univariate**  **OR (95% CI)** | **Demographic variables**  **OR (95% CI)** | **+ Clinical variables**  **OR (95% CI)** | **+ Spirometry variables**  **OR (95% CI)** | **+ CXR variables**  **OR (95% CI)** |
| --- | --- | --- | --- | --- | --- |
| Male sex | 0.8 (0.4, 1.4) | 0.9 (0.4, 1.8) | 0.9 (0.4, 1.8) | 0.8 (0.4, 1.7) | 0.9 (0.4, 2) |
| Age (yrs) | 1 (1, 1) | 1 (1, 1.1) | 1 (1, 1.1) | **1 (1, 1.1)** | **1 (1, 1.1)** |
| Maximum education level > primary school | 1 (0.6, 1.9) | 1.1 (0.6, 2.1) | 1.3 (0.7, 2.7) | 1.6 (0.7, 3.4) | 1.6 (0.7, 3.5) |
| Positive TB microbiology* | 1.1 (0.6, 2.3) | 1.2 (0.6, 2.7) | 1.4 (0.7, 3.2) | 1.2 (0.6, 2.7) | 1.2 (0.6, 2.8) |
| HIV status (n=403) - Negative | 1.0 | 1.0 | 1.0 | 1.0 | 1.0 |
| - Positive, CD4 ≥200 | **0.5 (0.3, 1)** | **0.5 (0.2, 0.9)** | 0.5 (0.3, 1) | **0.5 (0.2, 1)** | **0.5 (0.2, 1)** |
| - Positive, CD4<200 | **0.4 (0.2, 1)** | **0.4 (0.2, 0.8)** | **0.4 (0.2, 0.9)** | 0.4 (0.2, 1) | 0.4 (0.2, 1) |
| Ever smoked | 0.6 (0.3, 1.1) | **0.4 (0.2, 1)** | **0.4 (0.2, 0.9)** | 0.5 (0.2, 1.1) | 0.5 (0.2, 1.1) |
| Main fuel - Charcoal | 1.0 | 1.0 | 1.0 | 1.0 | 1.0 |
| - Electricity | 0.3 (0, 1.5) | 0.3 (0, 1.6) | 0.4 (0, 2) | 0.4 (0, 2.1) | 0.4 (0, 2) |
| - Wood | 1.1 (0.4, 2.5) | 0.8 (0.3, 1.9) | 0.7 (0.3, 1.9) | 0.8 (0.3, 2.2) | 0.8 (0.3, 2.2) |
| Poorest 2 SES quintiles | 1.3 (0.7, 2.4) | 1.4 (0.7, 2.8) | 1.5 (0.7, 3.2) | 1.7 (0.8, 3.6) | 1.6 (0.8, 3.6) |
| Weekly cough | 1.9 (0.3, 9.1) |  | 0.5 (0.1, 2.8) | 1 (0.1, 7.1) | 0.9 (0.1, 7.1) |
| Weekly breathlessness | **6.1 (1.9, 19.6)** |  | **5.6 (1.5, 21.4)** | **6.6 (1.8, 26.4)** | **10.2 (2.4, 50)** |
| Limited walking pace | 1.5 (0.8, 2.8) |  | 0.8 (0.4, 1.8) | 0.8 (0.4, 1.9) | 0.9 (0.4, 2) |
| Limitation of activities | **2.1 (1.2, 3.9)** |  | **2 (1, 4.2)** | 1.8 (0.9, 3.9) | 1.8 (0.8, 3.9) |
| BMI (kg/m^2^) median | 1 (0.9, 1.1) |  | 1 (0.9, 1.1) | 1 (0.9, 1.1) | 1 (0.9, 1.1) |
| FEV_1_ 10% predicted | 0.9 (0.7, 1) |  |  | 0.8 (0.5, 1.3) | 0.8 (0.5, 1.3) |
| FVC 10% predicted | 0.9 (0.8, 1.1) |  |  | 1.2 (0.7, 2) | 1.1 (0.6, 1.8) |
| Pattern  - Normal | 1.0 |  |  | 1.0 | 1.0 |
| - Obstruction | 1.4 (0.6, 3) |  |  | 0.7 (0.2, 2.6) | 0.8 (0.2, 2.9) |
| - Low FVC | 1.7 (0.8, 3.3) |  |  | 1.3 (0.4, 3.6) | 1.1 (0.4, 3.2) |
| Lobar destruction | 2.4 (0.5, 9.4) |  |  |  | 1.5 (0.2, 8.5) |
| Ring & tramline markings | 0.8 (0.4, 1.6) |  |  |  | 0.8 (0.4, 1.7) |
| ≥10% Residual consolidation | 0.6 (0.1, 2) |  |  |  | 0.2 (0, 1) |
| ≥5% Residual cavitation | 1.1 (0.2, 3.5) |  |  |  | 0.8 (0.1, 3.2) |

Table D: Univariate and multivariable associations between pre-specified parameters and chronic respiratory symptoms, in logistic regression models, with no variable reduction (n=368)

| **Outcome: Chronic respiratory symptoms / functional limitation** | **Univariate**  **OR (95% CI)** | **Demographic variables**  **OR (95% CI)** | **+ Clinical variables**  **OR (95% CI)** | **+ Spirometry variables**  **OR (95% CI)** | **+ CXR variables**  **OR (95% CI)** |
| --- | --- | --- | --- | --- | --- |
| Male sex | 0.8 (0.5, 1.5) | 0.6 (0.3, 1.2) | 0.5 (0.2, 1) | 0.5 (0.2, 1.3) | 0.5 (0.2, 1.3) |
| Age (yrs) | 1 (1, 1.1) | **1 (1, 1.1)** | **1 (1, 1.1)** | 1 (1, 1.1) | 1 (1, 1.1) |
| Maximum education level > primary school | 0.6 (0.3, 1) | 0.7 (0.4, 1.4) | 0.9 (0.5, 1.9) | 1 (0.5, 2.1) | 0.9 (0.4, 2.1) |
| Positive TB microbiology* | 0.9 (0.5, 1.8) | 0.7 (0.4, 1.5) | 0.9 (0.4, 2) | 0.9 (0.4, 2.2) | 0.9 (0.4, 2.2) |
| HIV status (n=403) - Negative | 1.0 | 1.0 | 1.0 | 1.0 | 1.0 |
| - Positive, CD4 ≥200 | 0.5 (0.3, 1) | **0.5 (0.2, 0.9)** | 0.6 (0.3, 1.2) | 0.5 (0.2, 1.1) | 0.5 (0.2, 1.1) |
| - Positive, CD4<200 | 0.7 (0.3, 1.4) | 0.6 (0.3, 1.2) | 0.7 (0.3, 1.4) | 0.6 (0.2, 1.5) | 0.6 (0.2, 1.5) |
| Ever smoked | 1.3 (0.7, 2.4) | 1.4 (0.7, 2.9) | 1.4 (0.6, 3) | 1.4 (0.6, 3.4) | 1.5 (0.6, 3.6) |
| Main fuel - Charcoal | 1.0 | 1.0 | 1.0 | 1.0 | 1.0 |
| - Electricity | - | - | - | - | - |
| - Wood | 1.4 (0.6, 3.1) | 0.8 (0.3, 1.9) | 0.6 (0.2, 1.7) | 1.1 (0.4, 3.1) | 1.1 (0.4, 3) |
| Poorest 2 SES quintiles | 1.4 (0.8, 2.5) | 1.1 (0.6, 2.2) | 1.1 (0.5, 2.3) | 1 (0.4, 2.1) | 1 (0.4, 2.3) |
| Weekly cough | 3.4 (0.6, 15.6) |  | 0.8 (0.1, 4.3) | 0.7 (0.1, 5.5) | 0.9 (0.1, 7.4) |
| Weekly breathlessness | 25.7 (6.5, 170.5) |  | **18 (4, 133.2)** | **18.7 (3.9, 144.9)** | **16.7 (3.3, 133.4)** |
| Limited walking pace | **3.6 (2, 6.6)** |  | 1.2 (0.6, 2.4) | 1.1 (0.5, 2.4) | 1.1 (0.5, 2.4) |
| Limitation of activities | **6.4 (3.2, 13.8)** |  | **3.8 (1.8, 8.4)** | **4.3 (1.9, 10.6)** | **4.2 (1.8, 10.3)** |
| BMI (kg/m^2^) median | 0.9 (0.8, 1) |  | **0.9 (0.8, 1)** | 0.9 (0.8, 1) | 0.9 (0.8, 1) |
| FEV_1_ 10% predicted | 0.8 (0.7, 0.9) |  |  | **0.5 (0.3, 0.8)** | **0.5 (0.3, 0.9)** |
| FVC 10% predicted | 0.9 (0.7, 1.1) |  |  | 1.8 (1, 3.2) | **1.8 (1, 3.3)** |
| Pattern  - Normal | 1.0 |  |  | 1.0 | 1.0 |
| - Obstruction | 2.1 (1, 4.4) |  |  | 0.3 (0.1, 1.2) | 0.3 (0.1, 1.1) |
| - Low FVC | 1.4 (0.6, 2.7) |  |  | 0.7 (0.2, 2) | 0.7 (0.2, 2.2) |
| Lobar destruction | 2.2 (0.5, 8.7) |  |  |  | 0.9 (0.1, 5.6) |
| Ring & tramline markings | 0.9 (0.5, 1.7) |  |  |  | 0.8 (0.3, 1.7) |
| ≥10% Residual consolidation | **3.5 (1.4, 8.3)** |  |  |  | 1.8 (0.5, 5.8) |
| ≥5% Residual cavitation | 2.8 (0.9, 7.8) |  |  |  | 1.3 (0.3, 5.2) |

Cells with “-“ indicate that parameter was not estimable

Table E: Univariate and multivariable associations between pre-specified parameters and self-reported severe financial impact of disease, in logistic regression models, with no variable reduction(n=368)

| **Outcome: Severe financial impact** | **Univariate**  **OR (95% CI)** | **Demographic variables**  **OR (95% CI)** | **+ Clinical variables**  **OR (95% CI)** | **+ Spirometry variables**  **OR (95% CI)** | **+ CXR variables**  **OR (95% CI)** |
| --- | --- | --- | --- | --- | --- |
| Male sex | 1.2 (0.7, 2.5) | 1 (0.4, 2.1) | 0.9 (0.4, 2.1) | 0.7 (0.3, 1.8) | 0.7 (0.3, 1.8) |
| Age (yrs) | **1.1 (1, 1.1)** | **1.1 (1, 1.1)** | **1.1 (1, 1.1)** | **1.1 (1, 1.1)** | **1.1 (1, 1.1)** |
| Maximum education level > primary school | **0.5 (0.3, 0.9)** | 0.9 (0.5, 1.8) | 0.9 (0.5, 1.8) | 0.9 (0.5, 1.9) | 0.9 (0.4, 1.9) |
| Positive TB microbiology* | 0.7 (0.4, 1.4) | 0.8 (0.4, 1.6) | 0.8 (0.4, 1.6) | 0.7 (0.3, 1.4) | 0.6 (0.3, 1.4) |
| HIV status (n=403) - Negative | 1.0 | 1.0 | 1.0 | 1.0 | 1.0 |
| - Positive, CD4 ≥200 | 1.8 (0.9, 4) | 1.4 (0.7, 2.9) | 1.5 (0.7, 3.2) | 1.6 (0.7, 3.8) | 1.7 (0.7, 4) |
| - Positive, CD4<200 | **2.7 (1.3, 6)** | 1.9 (0.9, 4.1) | 2 (0.9, 4.5) | 2.3 (1, 5.6) | 2.5 (1, 6.2) |
| Ever smoked | 2 (1.1, 3.6) | 1.9 (1, 4) | 2 (1, 4.3) | 2.2 (1, 5) | 2.2 (1, 5.1) |
| Main fuel - Charcoal | 1.0 | 1.0 | 1.0 | 1.0 | 1.0 |
| - Electricity | 0.4 (0, 1.8) | 0.4 (0, 2.1) | 0.5 (0, 2.6) | 0.6 (0, 3.7) | 0.7 (0, 4) |
| - Wood | 1.4 (0.6, 3.2) | 0.8 (0.3, 1.8) | 0.7 (0.3, 1.8) | 0.8 (0.3, 2.2) | 0.8 (0.3, 2.2) |
| Poorest 2 SES quintiles | **2.5 (1.4, 4.6)** | **2.3 (1.1, 4.5)** | 2 (1, 4.1) | **2.3 (1.1, 5)** | **2.4 (1.1, 5.2)** |
| Weekly cough | 2 (0.3, 9.6) |  | 1.2 (0.2, 6.7) | 2.1 (0.2, 14.8) | 2.2 (0.2, 18.1) |
| Weekly breathlessness | 1.4 (0.6, 3.2) |  | 0.3 (0, 2) | 0.4 (0, 2.6) | 0.3 (0, 2.3) |
| Limited walking pace | 1.1 (0.6, 2.2) |  | 0.6 (0.3, 1.4) | 0.6 (0.3, 1.5) | 0.6 (0.3, 1.5) |
| Limitation of activities | **1.9 (1.1, 3.6)** |  | **2.3 (1.1, 4.8)** | **2.3 (1.1, 5)** | **2.3 (1, 5)** |
| BMI (kg/m^2^) median | 0.9 (0.8, 1.1) |  | 0.9 (0.8, 1.1) | 0.9 (0.8, 1.1) | 0.9 (0.8, 1.1) |
| FEV_1_ 10% predicted | 1 (0.8, 1.2) |  |  | 0.9 (0.5, 1.4) | 0.9 (0.5, 1.5) |
| FVC 10% predicted | 1.1 (0.9, 1.3) |  |  | 1.2 (0.7, 1.9) | 1.2 (0.7, 2) |
| Pattern  - Normal | 1.0 |  |  | 1.0 | 1.0 |
| - Obstruction | 1 (0.4, 2.3 |  |  | 0.6 (0.1, 2.2) | 0.5 (0.1, 2.2) |
| - Low FVC | 0.8 (0.3, 1.7) |  |  | 0.9 (0.3, 2.8) | 1 (0.3, 3.1) |
| Lobar destruction | 1.4 (0.2, 6.1) |  |  |  | 2.1 (0.2, 12.7) |
| Ring & tramline markings | 1 (0.5, 1.9) |  |  |  | 1 (0.4, 2.2) |
| ≥10% Residual consolidation | 1.3 (0.4, 3.5) |  |  |  | 1.6 (0.4, 5.3) |
| ≥5% Residual cavitation | 1.1 (0.3, 3.7) |  |  |  | 0.7 (0.1, 3.2) |
