# Supplementary material for: Screening for post-TB lung disease at TB treatment completion: Are symptoms sufficient?
Source: PLOS Glob Public Health. 2024 Jan 29;4(1):e0002659. doi: 10.1371/journal.pgph.0002659 (PMC10824425; doi:10.1371/journal.pgph.0002659)
Supplement: S5 Text — (DOCX) [file pgph.0002659.s005.docx]

S5 Table: Adjusted odds ratios estimating associations between pre-specified parameters and outcomes, by logistic regression model, including all pre-specified covariates with no variable reduction in microbiology confirmed TB participants only

|  | **Spirometry decline (n=54/227) OR (95% CI)** | **Health seeking (n=45/250) OR (95% CI)** | **Symptoms / limitation (n=68/250) OR (95% CI)** | **Severe financial impact (n=39/249) OR (95% CI)** |
| --- | --- | --- | --- | --- |
| Demographic parameters |  |  |  |  |
| Male sex | **0.2 (0.1, 0.6)** | 0.6 (0.2, 1.6) | 0.6 (0.3, 1.5) | 1.6 (0.5, 5.2) |
| Age (yrs) | **1.0 (1.0, 1.1)** | 1.0 (1.0, 1.1) | **1.0 (1.0, 1.1)** | **1.1 (1.0, 1.1)** |
| Maximum education level > primary school | 1.6 (0.7, 4) | 2.1 (0.8, 5.8) | 1.7 (0.8, 3.9) | 0.9 (0.4, 2.2) |
| HIV status (n=403)  - Negative  - Positive, CD4 >=200  - Positive, CD4<200 | 1.0  **0.3 (0.1, 0.8)**  0.5 (0.2, 1.3) | 1.0  **0.4 (0.2, 1.0)**  0.5 (0.2, 1.2) | 1.0  **0.5 (0.2, 1.0)**  0.4 (0.2, 1.0) | 1.0  1.7 (0.7, 4.4)  2.5 (0.9, 7.2) |
| Ever smoked | 2.0 (0.8, 5.4) | 0.6 (0.2, 1.6) | 1.1 (0.5, 2.6) | 1.9 (0.8, 5.0) |
| Main fuel*  - Charcoal  - Electricity  - Wood | 1.0  2.0 (0.8, 5.4)  0.2 (0.6, 2.0) | 1.0  -  0.6 (0.2, 2.1) | 1.0  -  1.1 (0.4, 3) | 1.0  1.0 (0.1, 7.5)  1.0 (0.3, 3.2) |
| Poorest 2 SES quintiles | **3.6 (1.4, 9.3)** | 1.9 (0.8, 4.8) | 1.3 (0.6, 2.8) | 2.0 (0.8, 5.1) |
| Clinical parameters at TB treatment completion | | | | |
| BMI (kg/m^2^) median | 1.1 (1.0, 1.3) | 1.1 (1.0, 1.3) | 1.0 (0.9, 1.1) | 1.0 (0.9, 1.2) |
| Weekly cough | 4.1 (0.2, 52.1) | 0.7 (0.1, 5.3) | 0.5 (0.1, 3.6) | 1.5 (0.1, 13.8) |
| Weekly breathlessness | **27.9 (3.3, 337.2)** | **27 (4.5, 206.2)** | **16.2 (2.9, 140.3)** | 0.5 (0.1, 13.8) |
| Limited walking | 0.7 (0.2, 2.1) | 0.5 (0.2, 1.4) | 1.0 (0.4, 2.3) | 0.4 (0.2, 1.2) |
| Limitation of activities | 1.9 (0.8, 4.7) | **2.7 (1.1, 6.5)** | **3.6 (1.7, 8.0)** | **4.4 (1.7, 11.8)** |
| Spirometry at TB treatment completion | | | | |
| 10% larger FEV_1_ % predicted | 0.6 (0.3, 1.1) | 0.7 (0.4, 1.2) | **0.5 (0.3, 0.9)** | 0.9 (0.5, 1.6) |
| 10% larger FVC % predicted | **2.3 (1.2, 4.6)** | 1.0 (0.6, 1.9) | **1.4 (0.8, 2.4)** | 1.1 (0.6, 2.1) |
| Spirometry pattern  - Normal  - Obstruction  - Low FVC | **1.0**  **0.2 (0.0, 0.9)**  **0.5 (0.1, 2.0)** | 1.0  0.6 (0.1, 3.1)  0.9 (0.2, 3.3) | 1.0  0.4 (0.1, 1.8)  0.9 (0.3, 2.7) | 1.0  0.6 (0.1, 3.3)  1.3 (0.3, 4.9) |
| Imaging at TB treatment completion | | | | |
| Lobar destruction* | - | 1.9 (0.2, 13.8) | 0.7 (0.1, 4.7) | 2.7 (0.3, 18.9) |
| Ring & tramline markings | 0.9 (0.4, 2.1) | 1.0 (0.4, 2.4) | 1.0 (0.5, 2.2) | 1.1 (0.4, 2.6) |
| ≥10% Residual consolidation | 0.2 (0, 1.6) | 0.1 (0.0, 1.0) | 0.3 (0.1, 1.1) | 1.1 (0.2, 4.7) |
| ≥5% Residual cavitation | 1.0 (0.1, 6.5) | 0.9 (0.1, 4.2) | 0.9 (0.2, 3.4) | 0.7 (0.1, 3.3) |

*Variables where ‘-‘ given are unstable and OR not interpretable
