# Supplementary material for: Screening for post-TB lung disease at TB treatment completion: Are symptoms sufficient?
Source: PLOS Glob Public Health. 2024 Jan 29;4(1):e0002659. doi: 10.1371/journal.pgph.0002659 (PMC10824425; doi:10.1371/journal.pgph.0002659)
Supplement: S6 Text — (DOCX) [file pgph.0002659.s006.docx]

S6 Table: Multivariable associations (OR (95% CI) between pre-specified parameters and outcomes, in penalised regression models

|  | **Spirometry decline (n=71/305)** | **Health seeking**  **(n=62/368)** | **Symptoms / limitation (n=73/368)** | **Severe financial impact (n=62/368)** |
| --- | --- | --- | --- | --- |
| Male sex | **0.3 (0.1, 0.7)** | 0.9 (0.4, 2) | 0.6 (0.2, 1.3) | 0.7 (0.3, 1.8) |
| Age (yrs) | 1 (1, 1.1) | **1 (1, 1.1)** | **1 (1, 1.1)** | **1.1 (1, 1.1)** |
| Maximum education level > primary school | 1.3 (0.6, 2.7) | 1.6 (0.8, 3.5) | - | 0.9 (0.4, 1.9) |
| Positive TB microbiology* | - | 1.2 (0.6, 2.8) | - | 0.6 (0.3, 1.4) |
| HIV status (n=403) - Negative | 1.0 | 1.0 | - | 1.0 |
| - Positive, CD4 ≥200 | **0.3 (0.1, 0.7)** | **0.5 (0.2, 1)** | - | 1.7 (0.8, 4) |
| - Positive, CD4<200 | 0.5 (0.2, 1.1) | **0.4 (0.2, 1)** | - | **2.5 (1, 6.2)** |
| Ever smoked | 1.3 (0.6, 3) | 0.5 (0.2, 1.1) | 1.6 (0.7, 3.6) | 2.2 (1, 5.1) |
| Main fuel - Charcoal | 1.0 | 1.0 | - | **1.0** |
| - Electricity | 3.6 (0.9, 13.8) | 0.4 (0, 2) | - | 0.7 (0, 3.9) |
| - Wood | 0.6 (0.2, 1.7) | 0.8 (0.3, 2.2) | - | 0.8 (0.3, 2.2) |
| Poorest 2 SES quintiles | **2.8 (1.2, 6.6)** | 1.6 (0.8, 3.6) | - | **2.4 (1.1, 5.2)** |
| Weekly cough | 3.4 (0.1, 47.5) | - | - | 2.2 (0.2, 17.8) |
| Weekly breathlessness | **20.4 (3, 196.5)** | **10.2 (2.5, 48.4)** | **16.6 (3.6, 122.7)** | 0.2 (0, 2.3) |
| Limited walking pace | 0.4 (0.2, 1.1) | 0.9 (0.4, 2) | 1 (0.5, 2.1) | 0.6 (0.3, 1.5) |
| Limitation of activities | 2.1 (1, 4.6) | 1.8 (0.8, 3.9) | **4.2 (1.8, 10)** | **2.3 (1, 5)** |
| BMI (kg/m^2^) median | **1.2 (1, 1.3)** | 1 (0.9, 1.1) | 0.9 (0.8, 1) | 0.9 (0.8, 1.1) |
| FEV_1_ 10% predicted | 1 (0.6, 1.7) | 0.8 (0.5, 1.3) | 0.9 (0.7, 1.1) | 0.9 (0.5, 1.5) |
| FVC 10% predicted | **1.7 (1, 3)** | 1.1 (0.6, 1.8) | **-** | 1.2 (0.7, 2) |
| Pattern  - Normal | 1.0 | 1.0 | **-** | 1.0 |
| - Obstruction | 0.6 (0.1, 2.4) | 0.7 (0.2, 2.8) | - | 0.5 (0.1, 2.2) |
| - Low FVC | 0.7 (0.2, 2.7) | 1.1 (0.4, 3.2) | - | 1 (0.3, 3.1) |
| Lobar destruction | - | 1.5 (0.2, 8.2) | 1.3 (0.2, 7.7) | 2.1 (0.2, 12.7) |
| Ring & tramline markings | 0.8 (0.4, 1.9) | 0.8 (0.4, 1.7) | 0.7 (0.3, 1.5) | - |
| ≥10% Residual consolidation | 0.2 (0, 1.2) | 0.2 (0, 1) | 1.6 (0.5, 4.7) | 1.6 (0.4, 5.3) |
| ≥5% Residual cavitation | 1.7 (0.2, 11.3) | 0.8 (0.1, 3.2) | 1.2 (0.3, 4.6) | 0.7 (0.1, 3.2) |

Data for death not shown, due to model instability
