# Supplementary material for: Screening for post-TB lung disease at TB treatment completion: Are symptoms sufficient?
Source: PLOS Glob Public Health. 2024 Jan 29;4(1):e0002659. doi: 10.1371/journal.pgph.0002659 (PMC10824425; doi:10.1371/journal.pgph.0002659)
Supplement: S7 Text — (DOCX) [file pgph.0002659.s007.docx]

S7 Table: Variable importance values for prediction of outcome, using full parent data set (Higher value indicates greater importance in predicting outcome)

| **Predictors** | **Death**  **(n=11/405)** | **Spirometry decline**  **(n=71/305)** | **Health seeking**  **(n=62/368)** | **Symptoms / limitation**  **(n=73/368)** | **Severe financial impact**  **(n=62/368)** |
| --- | --- | --- | --- | --- | --- |
| Age | 12.39 | . | . | . | 71.79 |
| Male gender | . | 32.95 | 11.17 | . | . |
| Education beyond primary school | 51.52 | . | . | . | . |
| Low SES | . | 18.77 | 32.25 | . | 42.62 |
| Financial impact of TB | . | . | . | . | 100 |
| Missing meals | 12.76 | . | . | . | . |
| Dissaving, by TB treatment completion | 14.81 | 13.18 | 4.48 | 4.53 | 13.19 |
| Interruptions of schooling | . | . | 19.99 | . | . |
| Microbiologically proven PTB | 40.36 | . | . | . | 2.41 |
| Illness duration | 3.36 | . | 29.93 | . | . |
| HIV positive, CD4 >=200 | 82.09 | . | . | . | 11.06 |
| HIV positive, CD4<200 | . | 2.44 | 15.92 | . | . |
| Ever smoker | 12.32 | . | 54.76 | . | 21.5 |
| Main fuel | 7.17 | . | 27.75 | . | . |
| BMI (kg/m3) | 25.45 | 21.11 | . | . | . |
| Heart rate | 51.3 | 2.58 | 79.73 | . | . |
| Tachycardia | 14.18 | . | . | . | . |
| Respiratory rate | . | . | 8.96 | . | 0.28 |
| Tachypnoea | 18.95 | . | . | . | . |
| Saturations | . | 5.46 | . | . | 34.09 |
| Hypoxia | 4.35 | . | 15.88 | . | . |
| 6MWD (m) | 18.14 | 11.35 | . | . | 43.55 |
| SGRQ activity score | . | . | . | 0.87 | . |
| SGRQ impact score | . | . | 33.68 | . | 19.35 |
| SGRQ activity score | 6.1 | 25.97 | 100 | 100 | . |
| SGRQ total score | . | . | . | 4.62 | . |
| Regular cough | . | . | . | . | 1.69 |
| Regular SOB | 10.88 | . | 1.43 | . | . |
| Regular sputum | 100 | 5.18 | 5.29 | 15.33 | . |
| Regular wheeze | 34.92 | . | 18.02 | . | . |
| Any regular symptoms | 8.62 | . | . | . | . |
| Any weekly symptoms | 16.04 | 19.24 | 47.93 | 53.27 | 7.71 |
| Good days in past 3m | . | . | 38.55 | . | . |
| Limitation of activities | 49.16 | . | . | 22.85 | . |
| Problems relating to chest | 27.82 | . | 40.51 | . | 10.3 |
| SOB on incline | 92.09 | . | 26.13 | . | 11.22 |
| Slow on housework | . | 8.57 | . | 16.76 | 3.4 |
| Slow on hurrying | 23.87 | . | 23.11 | . | . |
| Limited walking pace | . | . | 3.67 | . | . |
| Difficulty heavy lifting | 25.45 | . | 48.42 | . | . |
| Difficulty farming | 8.48 | . | 12.59 | . | . |
| Difficulty lifting things | . | . | 3.36 | . | . |
| Mild SOB | . | . | 24.57 | . | . |
| Severe SOB | 3.9 | . | . | 6.98 | 37.75 |
| Self reported frailty | 12.1 | . | . | . | . |
| Exhaustion | 0.54 | . | . | . | . |
| EQ5D3L anxiety score | . | . | . | . | 18.96 |
| EQ5D3L mobility score | . | . | . | 13.1 | . |
| EQ5D3L pain score | . | . | 6.34 | . | . |
| EQ5D3L self care score | 9.37 | . | . | . | . |
| EQ5D3L activity score | 17.74 | 8.64 | . | . | . |
| EQ5D3L VAS score | . | . | 0.45 | . | 0.33 |
| % predicted FVC | . | 100 | . | . | . |
| FEV/FVC ratio | . | . | . | 17.54 | . |
| ≥10% Residual consolidation | 35.31 | . | 55.32 | . | . |
| % abnormal parenchyma | . | 76.17 | . | 21.01 | . |
| % atelectasis | 22.7 | . | 38.81 | . | . |
| % parenchymal banding | 22.01 | . | 0.4 | . | . |
| Ring and tramline | 35.06 | . | . | . | . |
| Pleural pathology | 24.51 | . | . | . | . |
| Hyperexpansion | . | . | . | . | 2.91 |
| Nodules | . | 10.59 | . | . | . |
