# Supplementary material for: Screening for post-TB lung disease at TB treatment completion: Are symptoms sufficient?
Source: PLOS Glob Public Health. 2024 Jan 29;4(1):e0002659. doi: 10.1371/journal.pgph.0002659 (PMC10824425; doi:10.1371/journal.pgph.0002659)
Supplement: S8 Text — (DOCX) [file pgph.0002659.s008.docx]

S8 Table: Models constructed for each outcome, from full parent data set

|  | **Outcome variables, OR (95% CI)** | | | | |
| --- | --- | --- | --- | --- | --- |
| **Predictors, at TB treatment completion** | **Death***  **(n=11/405)** | **Spirometry decline**  **(n=71/305)** | **Health seeking**  **(n=62/368)** | **Symptoms / limitation**  **(n=73/368)** | **Severe financial impact**  **(n=62/368)** |
| Age | . | . | . | . | **1.1 (1, 1.1)** |
| Male gender | . | 0.5 (0.2, 1.2) | 0.5 (0.2, 1.4) | . | . |
| Education beyond primary school | 3.8 (0.7, 32.5) | . | . | . | . |
| Low SES | . | 1.6 (0.8, 3.2) | 2.1 (0.9, 5.1) | . | **0.4 (0.2, 0.9)** |
| Financial impact of TB | . | . | . | . | 2.6 (1.3, 5.4) |
| Dissaving incurred | . | 0.6 (0.3, 1.3) | 1.5 (0.6, 4.1) | 0.5 (0.2, 1) | 1.9 (0.6, 7) |
| Interruptions of schooling | . | . | 1.6 (0.6, 4.2) | . | . |
| Microbiologically proven PTB | 0.3 (0.1, 1.3) | . | . | . | 1 (0.4, 2.4) |
| Illness duration prior to treatment | . | . | 1 (1, 1) | . | . |
| HIV positive, CD4 >=200 | 8.6 (1.3, 173) | 0.6 (0.3, 1.5) | 0.9 (0.3, 2.6) | . | 1.9 (0.6, 5.5) |
| HIV positive, CD4<200 | 1.3 (0.1, 30.8) | 0.6 (0.2, 1.3) | 0.6 (0.3, 1.5) | . | 1.3 (0.5, 3.5) |
| Ever smoker | . | . | 0.5 (0.2, 1.3) | . | 1.8 (0.8, 4) |
| Main fuel | . | . | 0.2 (0, 1.8) | . | . |
| BMI (kg/m3) | 0.8 (0.6, 1.1) | 1.1 (1, 1.2) | . | . | . |
| Heart rate | 1.1 (1, 1.1) | 1 (1, 1) | 1 (0.9, 1) | . | . |
| Respiratory rate | . | . | 1.1 (1, 1.3) | . | 0.9 (0.8, 1) |
| Saturations | . | 0.9 (0.7, 1.1) | . | . | **0.7 (0.6, 1)** |
| Hypoxia | . | . | 2.3 (0.6, 8.2) | . | . |
| 6MWD (m) | . | 1 (1, 1) | . | . | **1 (1, 1)** |
| SGRQ activity score | . | . | . | 1 (1, 1.1) | . |
| SGRQ impact score | . | . | 1.1 (1, 1.2) | . | **1 (1, 1.1)** |
| SGRQ activity score | . | 1 (1, 1.1) | 1 (1, 1.1) | 1 (1, 1.1) | . |
| SGRQ total score | . | . | . | 1 (0.9, 1.1) | . |
| Regular cough | . | . | . | . | 0.5 (0.2, 1.2) |
| Regular SOB | . | . | 1.6 (0.4, 6.9) | . | . |
| Regular sputum | . | 1.7 (0.7, 4) | 1.1 (0.4, 3.2) | 1.6 (0.7, 3.6) | . |
| Regular wheeze | 0.3 (0, 6.7) | . | 1.5 (0.3, 6.7) | . | . |
| Any weekly symptoms | . | 3 (0.6, 16.3) | 1.7 (0.3, 10.7) | **4 (1, 17)** | 0.4 (0, 2.4) |
| Good days in past 3m | . | . | 0.2 (0, 1.4) | . | . |
| Limitation of activities | 1.2 (0.2, 6.9) | . | . | 2.3 (0.7, 7.2) | . |
| Problems relating to chest | 2.4 (0, 85.9) | . | **0.1 (0, 0.6)** | . | 0.4 (0, 2.6) |
| SOB on incline | . | . | 1.2 (0.3, 4.5) | . | 1.6 (0.6, 4.1) |
| Slow on housework | . | 3.4 (0.8, 15.1) | . | 2.1 (0.6, 6.9) | 1.6 (0.4, 7.1) |
| Slow on hurrying | . | . | **0.2 (0.1, 0.8)** | . | . |
| Limited walking pace | . | . | 0.5 (0.1, 1.4) | . | . |
| Difficulty heavy lifting | 0.6 (0, 4.9) | . | 0.2 (0, 1) | . | . |
| Difficulty farming | . | . | 0.4 (0.1, 1.2) | . | . |
| Difficulty lifting things | . | . | 0.5 (0.2, 1.6) | . | . |
| Mild SOB | . | . | 2.7 (0.8, 10.3) | . | . |
| Severe SOB | . | . | . | 1.5 (0.5, 4.3) | **0.2 (0.1, 0.8)** |
| EQ5D3L anxiety score | . | . | . | . | 0.4 (0.2, 1) |
| EQ5D3L mobility score | . | . | . | 1.6 (0.7, 3.4) | . |
| EQ5D3L pain score | . | . | 1.3 (0.5, 3.1) | . | . |
| EQ5D3L activity score | . | **0.2 (0.1, 0.7)** | . | . | . |
| EQ5D3L VAS score | . | . | 1 (1, 1) | . | 1 (1, 1) |
| % predicted FVC | . | **1.1 (1, 1.1)** | . | . | . |
| FEV/FVC ratio | . | . | . | 1 (0.9, 1) | . |
| ≥10% Residual consolidation | . | . | 0.2 (0, 1) | . | . |
| % abnormal parenchyma | . | **0.9 (0.8, 1)** | . | 1 (1, 1.1) | . |
| % atelectasis | . | . | 1.1 (1, 1.2) | . | . |
| % parenchymal banding | . | . | 1.5 (0.7, 3) | . | . |
| Ring and tramline | 0.4 (0, 2.8) | . | . | . | . |
| Hyperexpansion | . | . | . | . | 0.4 (0.1, 1.3) |
| Nodules | . | 0.5 (0.2, 1.2) | . | . | . |

*Models for death include variables with Variable Importance Factor>25 only, where no convergence seen
