# Supplementary material for: Screening for post-TB lung disease at TB treatment completion: Are symptoms sufficient?
Source: PLOS Glob Public Health. 2024 Jan 29;4(1):e0002659. doi: 10.1371/journal.pgph.0002659 (PMC10824425; doi:10.1371/journal.pgph.0002659)

S9 Figure: ROC curves for logistic regression models for each outcome, using pre-specified predictor variable sets after variable reduction with penalised regression (Group 1: demographic only; Group 2: demographic plus clinical; Group 3: demographic, clinical, spirometry; Group 4: Demographic, clinical, spirometry and CXR)

A. Death

C. Unscheduled Resp visits

B. Spirometry decline

D. Symptoms / limitation

E. Severe financial impact


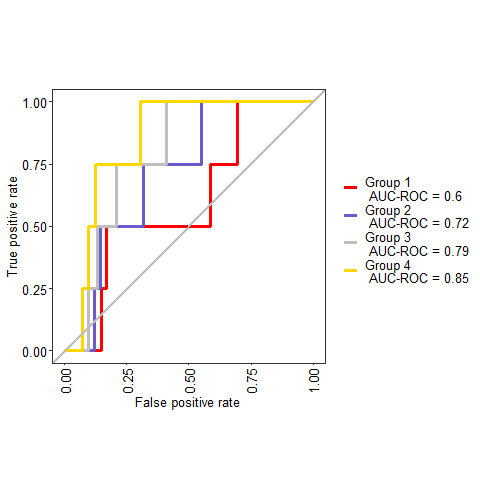

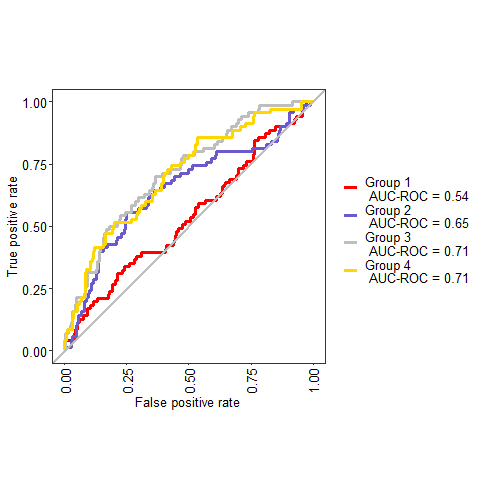

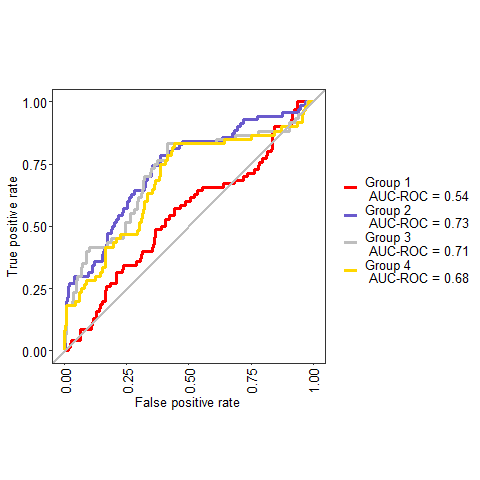

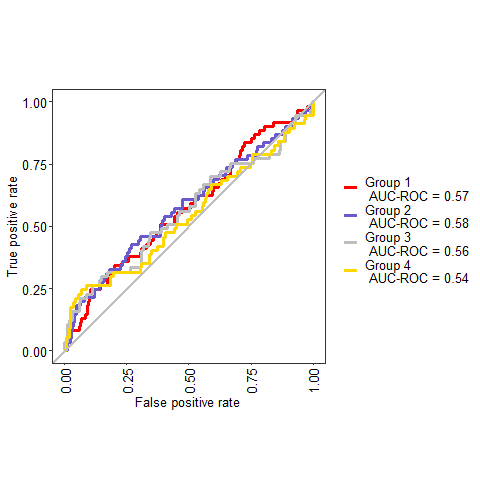

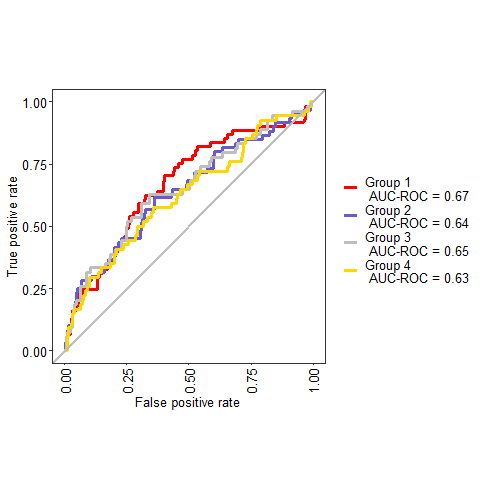

Supplement: S9 Text — (DOCX) [file pgph.0002659.s009.docx]
