# Supplementary material for: Screening for post-TB lung disease at TB treatment completion: Are symptoms sufficient?
Source: PLOS Glob Public Health. 2024 Jan 29;4(1):e0002659. doi: 10.1371/journal.pgph.0002659 (PMC10824425; doi:10.1371/journal.pgph.0002659)
Supplement: S10 Text — (DOCX) [file pgph.0002659.s010.docx]

S10 Table: Sensitivity, specificity, and predictive values of individual symptoms questions for each outcome

| Outcome | Symptom question | Sensitivity | Specificity | PPV | NPV |
| --- | --- | --- | --- | --- | --- |
| Death | Chest problems | 9.1% | 93.9% | 4.8% | 96.9% |
|  | Limited walking pace | 54.5% | 73.3% | 6.4% | 98.0% |
|  | Chest attacks | 63.6% | 69.0% | 6.4% | 98.3% |
|  | Regular cough | 72.7% | 64.7% | 6.5% | 98.6% |
|  | Interfering with work | 72.7% | 60.2% | 5.8% | 98.5% |
|  | Regular SOB | 81.8% | 55.9% | 5.8% | 98.9% |
|  | SOB on incline | 90.9% | 57.1% | 6.6% | 99.5% |
|  | Limitation of activities | 72.7% | 48.9% | 4.5% | 98.2% |
| Spirometry decline | Chest problems | 6.0% | 93.6% | 22.2% | 76.4% |
|  | Limited walking pace | 22.4% | 73.4% | 20.5% | 75.5% |
|  | Chest attacks | 32.8% | 71.6% | 26.2% | 77.6% |
|  | Regular cough | 38.8% | 65.6% | 25.7% | 77.7% |
|  | Interfering with work | 38.8% | 61.5% | 23.6% | 76.6% |
|  | Regular SOB | 44.8% | 58.7% | 25.0% | 77.6% |
|  | SOB on incline | 43.3% | 59.6% | 24.8% | 77.4% |
|  | Limitation of activities | 53.7% | 50.9% | 25.2% | 78.2% |
| Respiratory health seeking | Chest problems | 10.7% | 94.9% | 31.6% | 82.8% |
|  | Limited walking pace | 33.9% | 75.1% | 23.2% | 83.7% |
|  | Chest attacks | 50.0% | 72.7% | 28.9% | 86.8% |
|  | Regular cough | 51.8% | 66.8% | 25.7% | 86.2% |
|  | Interfering with work | 55.4% | 63.2% | 25.0% | 86.5% |
|  | Regular SOB | 64.3% | 60.5% | 26.5% | 88.4% |
|  | SOB on incline | 64.3% | 62.1% | 27.3% | 88.7% |
|  | Limitation of activities | 66.1% | 51.4% | 23.1% | 87.2% |
| Symptoms or activity limitation | Chest problems | 18.3% | 96.8% | 57.9% | 83.1% |
|  | Limited walking pace | 48.3% | 79.5% | 36.3% | 86.5% |
|  | Chest attacks | 60.0% | 76.3% | 37.9% | 88.8% |
|  | Regular cough | 55.0% | 68.7% | 29.7% | 86.4% |
|  | Interfering with work | 63.3% | 66.7% | 31.4% | 88.3% |
|  | Regular SOB | 66.7% | 62.2% | 29.9% | 88.6% |
|  | SOB on incline | 68.3% | 64.3% | 31.5% | 89.4% |
|  | Limitation of activities | 80.0% | 55.8% | 30.4% | 92.1% |
| Severe financial impact | Chest problems | 5.7% | 93.8% | 15.8% | 82.8% |
|  | Limited walking pace | 28.3% | 74.6% | 18.8% | 83.4% |
|  | Chest attacks | 32.1% | 69.5% | 17.9% | 83.2% |
|  | Regular cough | 39.6% | 64.8% | 18.9% | 83.8% |
|  | Interfering with work | 49.1% | 62.9% | 21.5% | 85.6% |
|  | Regular SOB | 45.3% | 57.0% | 17.9% | 83.4% |
|  | SOB on incline | 52.8% | 60.2% | 21.5% | 86.0% |
|  | Limitation of activities | 64.2% | 51.6% | 21.5% | 87.4% |
| Chest problems: Chest problem causes quite a lot of problems, or is the most important problem | | | | | |
| Limited walking pace: Walk slower than others / stop for rests | | | | | |
| Chest attacks: At least one severe / unpleasant attack of chest trouble in past 3m | | | | | |
| Regular cough: Cough for >= few days per month for past 3m | | | | | |
| Interfering with work: Chest trouble interferes with / made me stop work | | | | | |
| Regular SOB: Breathlessness for >= few days per month for past 3m | | | | | |
| SOB on incline: Usually SOB when walking up hills | | | | | |
| Limitation of activities: Chest stops me doing 1-2 things, most of the things I would like, or everything | | | | | |
