# Supplementary material for: Screening for post-TB lung disease at TB treatment completion: Are symptoms sufficient?
Source: PLOS Glob Public Health. 2024 Jan 29;4(1):e0002659. doi: 10.1371/journal.pgph.0002659 (PMC10824425; doi:10.1371/journal.pgph.0002659)
Supplement: S11 Text — (DOCX) [file pgph.0002659.s011.docx]

S11 Table: Performance of indicators on CXR at TB treatment completion, in screening for adverse patient outcomes

| Screening tool at TB treatment end | Outcome, in the year after treatment completion | Sensitivity | Specificity | PPV | NPV |  |
| --- | --- | --- | --- | --- | --- | --- |
| Any abnormal spirometry (Obstruction or low FVC pattern) | Death | 18.2% | 65.3% | 1.7% | 96.0% |  |
|  | Spirometry decline | 19.4% | 61.5% | 13.4% | 71.3% |  |
|  | Respiratory health seeking | 39.3% | 68.0% | 21.4% | 83.5% |  |
|  | Symptoms or activity limitation | 43.3% | 69.1% | 25.2% | 83.5% |  |
|  | Severe financial impact | 32.1% | 66.4% | 16.5% | 82.5% |  |
| Parenchymal pathology – combined patterns (≥90% absent parenchyma in ≥1 lung lobe) | Death | 0% | 97.0% | 0% | 96.7% |  |
|  | Spirometry decline | 0% | 96.3% | 0% | 75.8% |  |
|  | Respiratory health seeking | 5.4% | 97.6% | 33.3% | 82.3% |  |
|  | Symptoms or activity limitation | 5.0% | 97.6% | 33.3% | 81.0% |  |
|  | Severe financial impact | 3.8% | 97.3% | 22.2% | 83.0% |  |
| Cavitation (≥5% Residual cavitation, across whole lung) | Death | 9.1% | 94.8% | 5.6% | 96.9% |  |
|  | Spirometry decline | 3.0% | 94.5% | 14.3% | 76.0% |  |
|  | Respiratory health seeking | 5.4% | 95.2% | 20.0% | 82.0% |  |
|  | Symptoms or activity limitation | 10.0% | 96.4% | 40.0% | 81.6% |  |
|  | Severe financial impact | 5.7% | 95.3% | 20.0% | 83.0% |  |
| Bronchiectasis (Any ring & tramlines, at least moderate) | Death | 9.1% | 71.1% | 1.0% | 95.9% |  |
|  | Spirometry decline | 19.4% | 68.8% | 16.0% | 73.5% |  |
|  | Respiratory health seeking | 26.8% | 71.1% | 17.0% | 81.4% |  |
|  | Symptoms or activity limitation | 28.3% | 71.5% | 19.3% | 80.5% |  |
|  | Severe financial impact | 30.2% | 71.9% | 18.2% | 83.2% |  |
| Consolidation (≥10% Residual consolidation, across whole lung) | Death | 27.3% | 93.0% | 11.5% | 97.4% |  |
|  | Spirometry decline | 1.5% | 89.9% | 4.3% | 74.8% |  |
|  | Respiratory health seeking | 5.4% | 92.1% | 13.0% | 81.5% |  |
|  | Symptoms or activity limitation | 16.7% | 94.8% | 43.5% | 82.5% |  |
|  | Severe financial impact | 9.4% | 93.0% | 21.7% | 83.2% |  |
