# Supplementary material for: Screening for post-TB lung disease at TB treatment completion: Are symptoms sufficient?
Source: PLOS Glob Public Health. 2024 Jan 29;4(1):e0002659. doi: 10.1371/journal.pgph.0002659 (PMC10824425; doi:10.1371/journal.pgph.0002659)
Supplement: S12 Text — (DOCX) [file pgph.0002659.s012.docx]

S12 Table: Performance of question about limitation of activity and shortness of breath on hills at TB treatment completion, in screening for adverse patient outcomes among TB microbiology confirmed participants only

| Symptom question at TB treatment end | Outcome, in the year after treatment completion | Sensitivity | Specificity | PPV | NPV |
| --- | --- | --- | --- | --- | --- |
| Limitation of activities  (n=152/313, 48.6%) | Death | 60.0% | 50.8% | 2.3% | 98.5% |
|  | Spirometry decline | 68.9% | 54.1% | 26.4% | 80.0% |
|  | Respiratory health seeking | 68.2% | 54.4% | 25.2% | 88.3% |
|  | Symptoms or activity limitation | 82.2% | 58.4% | 31.1% | 93.5% |
|  | Severe financial impact | 68.4% | 54.4% | 21.8% | 90.2% |
| Breathlessness on hills  (n=131/313, 41.8%) | Death | 100.0% | 58.9% | 4.5% | 100.0% |
|  | Spirometry decline | 43.1% | 62.4% | 25.6% | 78.5% |
|  | Respiratory health seeking | 65.9% | 65.1% | 29.9% | 89.4% |
|  | Symptoms or activity limitation | 71.1% | 67.0% | 33.0% | 91.0% |
|  | Severe financial impact | 55.3% | 62.7% | 21.6% | 88.2% |
