# Supplementary material for: Screening for post-TB lung disease at TB treatment completion: Are symptoms sufficient?
Source: PLOS Glob Public Health. 2024 Jan 29;4(1):e0002659. doi: 10.1371/journal.pgph.0002659 (PMC10824425; doi:10.1371/journal.pgph.0002659)
Supplement: S13 Text — (DOCX) [file pgph.0002659.s013.docx]

S13 Table: Patient characteristics, stratified by presence or absence of limitation of activities at TB treatment completion

|  | **Total (n=405)** | **Limitation of activities at TB Rx end** | |  |
| --- | --- | --- | --- | --- |
|  |  | **Yes (n=205)** | **No (n=200)** | **P value** |
| Demographic | | | | |
| Age (yrs) (median, IQR) | 35 (28-41) | 34 (29-40) | 25 (27-41) | 0.499 |
| Male sex (n, %) | 275 (67.9%) | 133 (64.9%) | 142 (71.0%) | 0.187 |
| Positive TB microbiology (n, %)† | 313 (77.3%) | 152 (74.1%) | 161 (80.5%) | 0.127 |
| HIV status (n=403)  - Negative  - Positive, CD4<200  - Positive, CD4 >=200 | 159 (39.5)  102 (25.3%)  142 (35.2%) | 82 (40.2%)  53 (26.0%)  69 (33.8%) | 77 (38.7%)  49 (24.6%)  73 (36.7%) | 0.833 |
| Maximum education level > primary school (n, %) | 251 (62.0%) | 120 (58.5%) | 131 (65.5%) | 0.149 |
| Poorest 2 socioeconomic quintiles (n, %) | 107 (28.8%) | 59 (31.9%) | 48 (25.7%) | 0.185 |
| Ever smoked (n, %) | 120 (29.6%) | 62 (30.2%) | 58 (29.0%) | 0.784 |
| Main fuel (n, %)  - Charcoal  - Electricity  - Wood | 338 (83.5%)  21 (5.2%)  46 (11.4%) | 173 (84.4%)  8 (3.9%)  24 (11.7%) | 165 (82.5%)  13 (16.5%)  22 (11.0%) | 0.495 |
| BMI (kg/m^2^) median (IQR) | 20.5 (19.0 – 22.3) | 20.1 (18.7 – 21.9) | 20.8 (19.4 – 22.4) | 0.007 |
| Clinical | | | | |
| Weekly cough (n, %) | 11 (2.7%) | 10 (4.9%) | 1 (0.5%) | 0.007 |
| Weekly breathlessness (n, %) | 17 (4.2%) | 17 (8.3%) | 0 (0.0%) | <0.001 |
| Limited walking pace (n=403) (n, %) | 108 (26.8%) | 105 (51.2%) | 3 (1.5%) | <0.001 |
| Spiro | | | | |
| FEV_1_ % predicted (median, IQR) | 85.5 (73.2 – 98.3) | 83.5 (69.8 – 94.9) | 88.1 (77.8 – 100.8) | <0.001 |
| FVC % predicted (median, IQR) | 88.6 (78.8 – 98) | 86.5 (75.6 – 96.4) | 90.4 (81.2-99.7) | 0.002 |
| Pattern  - Missing  - Normal  - Obstruction  - Low FVC | 40  240 (65.8%)  52 (14.2%)  73 (20.0%) | 19  108 (58.1%)  46 (24.7%)  32 (17.2%) | 21  132 (73.7%)  27 (15.1%)  20 (11.2%) | 0.007 |
| CXR | | | | |
| Ring & tramline markings | 119 (29.5%) | 57 (27.8%) | 62 (31.3%) | 0.44 |
| Lobar destruction | 12 (3.0%) | 8 (3.9%) | 4 (2.0%) | 0.266 |
| ≥10% Residual consolidation | 31 (7.7%) | 22 (10.7%) | 9 (4.5%) | 0.02 |
| ≥5% Residual cavitation | 21 (5.2%) | 19 (9.3%) | 2 (1.0%) | <0.001 |
| Outcomes | | | | |
| Death | 11 (2.7%) | 8 (3.9%) | 3 (1.5%) | <0.001 |
| Spirometry decline | 71 (23.3%) | 37 (24.7%) | 34 (21.9%) | 0.573 |
| Health seeking | 62 (16.3%) | 40 (21.3%) | 22 (11.5%) | 0.01 |
| Symptoms/ limitation at 1yr | 73 (19.8%) | 58 (31.7%) | 15 (8.1%) | <0.001 |
| Severe financial impact | 62 (16.8%) | 38 (20.8%) | 24 (13.0%) | 0.046 |
